# Prey depletion as a threat to the world's large carnivores

**DOI:** 10.1098/rsos.160252

**Published:** 2016-08-03

**Authors:** Christopher Wolf, William J. Ripple

**Affiliations:** Global Trophic Cascades Program, Department of Forest Ecosystems and Society, Oregon State University, Corvallis, OR 97331, USA

**Keywords:** prey depletion, prey base, large carnivores, predator–prey, carnivore conservation

## Abstract

Large terrestrial carnivores are an ecologically important, charismatic and highly endangered group of species. Here, we assess the importance of prey depletion as a driver of large carnivore endangerment globally using lists of prey species for each large carnivore compiled from the literature. We consider spatial variation in prey endangerment, changes in endangerment over time and the causes of prey depletion, finding considerable evidence that loss of prey base is a major and wide-ranging threat among large carnivore species. In particular, the clouded leopard (*Neofelis nebulosa*), Sunda clouded leopard (*Neofelis diardi*), tiger (*Panthera tigris*), dhole (*Cuon alpinus*) and Ethiopian wolf (*Canis simensis*) all have at least 40% of their prey classified as threatened on the International Union for the Conservation of Nature (IUCN) Red List and, along with the leopard (*Panethra pardus*), all of these species except the Ethiopian wolf have at least 50% of their prey classified as declining. Of the 494 prey species in our analysis, an average of just 6.9% of their ranges overlap protected areas. Together these results show the importance of a holistic approach to conservation that involves protecting both large carnivores directly and the prey upon which they depend.

## 1. Introduction

Large terrestrial carnivores are an ecologically important group of species. Many of these species have significant direct effects on their prey, which can lead to additional indirect effects. For example, wolves (*Canis lupus*) may reduce elk (*Cervus canadensis*) numbers directly or may change their behaviour, leading to changes in plant species diversity, plant species abundance and songbird communities [1]. In Australia, dingoes (*Canis dingo*) can limit fox (*Vulpes vulpes*) populations, indirectly benefiting numerous small mammals, many of which are endangered [2]. In addition, large carnivores can provide important ecosystem services. For example, in West Africa, lion (*Panthera leo*) and leopard (*Panthera pardus*) declines coincided with an increase in the abundances of olive baboons (*Papio anubis*)—a species that can pose a threat to agricultural crops—and declines in the abundances of small ungulates and primates [3]. Large carnivores are also associated with important economic and social human benefits due to their role as one of the primary drivers of wildlife viewing tourism [4]. Tourism, including wildlife viewing, is especially important in the developing world where it is a significant or growing component of the Gross Domestic Product in 11 of the 12 countries that contain 80% of the world's poor [5]. Furthermore, many people derive value from knowing that large carnivores exist in the wild. This ‘existence value’ is independent of value associated with wildlife viewing or other wildlife ‘use’ [6].

Among mammals, the large carnivores form a highly endangered group, and the future of many of these species is uncertain [7]. Most of these species face multiple serious threats to their survival, 77% have declining populations and 61% are classified as threatened by the International Union for Conservation of Nature (IUCN) [7,8]. Large carnivores are often persecuted by humans due to conflict over livestock and shared prey [9]. Owing to their charisma, endangered status and ecological significance, large carnivores have received considerable research effort [7,10,11]. Although direct persecution and habitat change have received much of the attention, the loss of prey is another potentially important threat to large carnivores. Globally, little work has been done on assessing the importance of prey depletion as a threat to large carnivores [7]. Herein, we analyse prey depletion as a threat to large carnivore survival from a global, macroecological perspective.

Abundant terrestrial mammalian prey are required for the survival of large carnivores [12,13]. In fact there is a strong relationship between prey and carnivore abundance—approximately 10 000 kg of prey supports about 90 kg of large carnivore biomass, regardless of species [13]. When sufficient prey is unavailable, large carnivore populations will decline, possibly becoming locally extinct. This can be compounded by large carnivore conflicts with livestock, which increase as carnivores search for alternative food sources [14]. Similarly, when large carnivores are forced to range more widely in search of prey, they may face greater exposure to anthropogenic threats including mortality on roads [15] and direct persection in regions with high human population densities [9].

So far, research on prey depletion has generally focused on single carnivore species, with no comprehensive multi-species analysis. Research on tigers (*Panthera tigris*) suggests that prey depletion poses a major threat to their survival [16–18]. Snow leopards (*Panthera uncia*) have also received attention in terms of loss of prey. Snow leopards occur in environments with relatively low productivity, leading to naturally low amounts of prey. Thus, they are particularly vulnerable to declines in prey. The snow leopard's range is primarily located in central Asia, where wild ungulates are declining due to competition with domestic livestock [14]. In the Congo Basin rainforest, leopard abundances decline and diets shift towards smaller prey species in regions near human settlements due to exploitative competition with bushmeat hunters [19]. In Africa, poachers' snares that are used to catch prey species often also catch their predators because they are attracted to carcasses in snare lines, making the trapping of prey species both an indirect and direct threat to carnivores [20].

Motivated by the status of large carnivores and the potential for loss of prey to threaten their survival, we assessed the endangerment status and population trends of the prey of large carnivores. In addition, we considered how prey endangerment varies across time and space. Given the wide array of serious threats faced by mammal and other species, we hypothesized that prey endangerment status has deteriorated over time. We further hypothesized that the prey of large carnivores are less endangered in the developed world as recent research indicates that carnivores in Europe are currently experiencing a recovery [21]. This hypothesis is consistent with the status of large carnivores improving in areas where the status of their prey has improved.

## 2. Material and methods

For our analysis of prey depletion, we focused only on terrestrial hypercarnivores as classified by Hunter [22] with mean adult body masses above 15 kg [7]. The hypercarnivores, also called obligate meat-eaters, are members of the mammalian order Carnivora that are dependent on meat for their survival. That is, their diets consist of at least 70% meat [23]. They form a group of 17 species in the carnivore families Canidae, Felidae and Hyaenidae (table 1). The smallest species in the group are the dingo and Ethiopian wolf (*Canis simensis*) at 15 kg. The largest is the tiger at 161 kg [7].

**Table 1.** The 17 obligate meat-eating carnivores with masses at least 15 kg. ‘Status’ is the IUCN Red List conservation status (2008 or later): LC, least concern; NT, near threatened; VU, vulnerable; EN, endangered; CR, critically endangered [8]. None of these species has changed in endangerment status since 1996, except the dingo (which was not assessed at that time) and the leopard, which was classified as LC. Estimated current population sizes are from the IUCN Red List.

scientific name	common name	status	trend	family	population size
*Acinonyx jubatus*	cheetah	VU	decreasing	Felidae	6674
*Canis lupus*	grey wolf	LC	stable	Canidae	168 000–183 000
*Canis dingo*	dingo	VU	decreasing	Canidae	
*Canis rufus*	red wolf	CR	increasing	Canidae	<150
*Canis simensis*	Ethiopian wolf	EN	decreasing	Canidae	360–440
*Crocuta crocuta*	spotted hyena	LC	decreasing	Hyaenidae	27 000–47 000
*Cuon alpinus*	dhole	EN	decreasing	Canidae	4500–10 500
*Lycaon pictus*	African wild dog	EN	decreasing	Canidae	6600
*Lynx lynx*	Eurasian lynx	LC	stable	Felidae	
*Neofelis diardi*	Sunda clouded leopard	VU	decreasing	Felidae	4500
*Neofelis nebulosa*	clouded leopard	VU	decreasing	Felidae	
*Panthera leo*	lion	VU	decreasing	Felidae	18 726–31 395
*Panthera onca*	jaguar	NT	decreasing	Felidae	
*Panthera pardus*	leopard	VU	decreasing	Felidae	
*Panthera tigris*	tiger	EN	decreasing	Felidae	3159
*Panthera uncia*	snow leopard	EN	decreasing	Felidae	4080–6590
*Puma concolor*	puma	LC	decreasing	Felidae	

For our analysis, we used the endangerment status and trends of large carnivores' prey as assessed in the IUCN Red List database [8] to quantify prey depletion. The IUCN Red List is ‘widely recognized as the most comprehensive, objective global approach for evaluating the conservation status of plant and animal species’ [8]. The endangerment status of all mammals was assessed in 2008 or later, making the data fairly current. Throughout our analysis, we used prey endangerment status and population trends as proxies for abundance since species abundances are generally not available at this scale [24].

We constructed lists of the mammal prey species of each large carnivore to assess prey endangerment status using the single most comprehensive diet meta-analysis for each predator species (sources in the electronic supplementary material, table S1). These diet meta-analysis papers synthesize individual diet studies throughout a large carnivore's range. If multiple meta-analyses existed, we used the most recent one, provided it cited the earlier work. A few large carnivores, such as the Sunda clouded leopard (*Neofelis diardi*), have not been studied extensively, and lack diet meta-analyses. For these species, we searched Thomson Reuters' Web of Science database for articles using their scientific names and built prey lists based on confirmed prey species according to the literature [25]. In cases where a prey species group is listed as a genus, family or other group of species within a genus or family, we included all prey in the group with ranges that intersect the predator's range. We excluded broader groups of prey like mammalian orders. We also developed lists of preferred prey species by noting which, if any, prey species were described as preferred by the authors of the diet meta-analysis papers.

After constructing the prey lists, we performed a basic assessment of the status of large carnivores' prey by summarizing the IUCN Red List endangerment status and population trends of the prey species for each large carnivore separately. Specifically, we recorded the percentages of prey for each large carnivore in each endangerment and population trend category. We considered threatened species to be those categorized as vulnerable, endangered or critically endangered [26]. We used the ‘Major Threat(s)’ sections of the Red List species fact sheets to manually determine the major threats faced by threatened prey species (habitat change, hunting for meat, etc.). In addition, we obtained prey body masses from Jones *et al*. [27] when available.

We also split the large carnivore ranges by continent to better highlight spatial variation in prey endangerment. We summarized results for the predators with ranges overlapping each continent. For example, the Eurasian lynx (*Lynx lynx*) is included in the results for both Europe and Asia and the mountain lion (*Puma concolor*) in both North and South America. We then split the prey lists accordingly by constructing shorter prey lists corresponding to prey species overlapping each large carnivores' range within a given continent. To visualize prey endangerment spatially, we used a Geodesic Discrete Global Grid System (DGGS) that divides the earth's surface into hexagons, each with area approximately 12 500 km^2^ [28,29]. We retained only the hexagons whose centres occurred over land and not large bodies of water. For each hexagon, we determined the large carnivores and prey species with ranges that overlapped it, allowing us to map the proportion of endangered species across each large carnivore's range at the hexagon level. We used this mapping approach to emphasize prey species with smaller ranges and to reduce the impact of range map inaccuracies [30].

To assess changes in prey endangerment, we obtained corrected 1996 IUCN endangerment statuses from Hoffmann *et al.* [26]. For our analysis of changes in endangerment, we considered only prey species that were assessed in 1996. Mammal species endangerment information in the current IUCN Red List was last updated in 2008 or later, so we refer to current statuses as 2008 status when making comparisons across time.

We also considered overlap between prey species ranges and protected areas using the World Database of Protected Areas [31]. We used only protected areas with IUCN categories Ia, Ib, II, or III (the highest levels of protection) and polygon (rather than point) spatial information. We clipped each prey species range to these protected areas in order to determine total and percentage area overlap.

We conducted a literature search to assess the extent to which prey depletion has been considered in large carnivore conservation research. For each of the large carnivore species in our analysis, we searched Thomson Reuters' Web of Science database for articles with title including the carnivore's scientific or common name and topic ‘conservation’ [25]. After excluding articles not related to the large carnivore searched for, we classified the six most cited articles for each carnivore based on the extent to which ‘prey’ was mentioned into three categories: not mentioned at all, mentioned without reference to prey endangerment, status or depletion or mentioned in the context of prey status or depletion.

## 3. Results

Altogether, the prey lists contain 494 species, of which 123 species (25%) were threatened: 67 (14%) are classified as vulnerable, 44 (9%) endangered and 12 (2%) critically endangered (electronic supplementary material, table S2). Additionally, 37 species (7%) are data deficient. Overall, threatened large carnivores had more threatened prey, with an average of 27% of their prey threatened compared with 19% for non-threatened carnivores.

**Figure 1.**
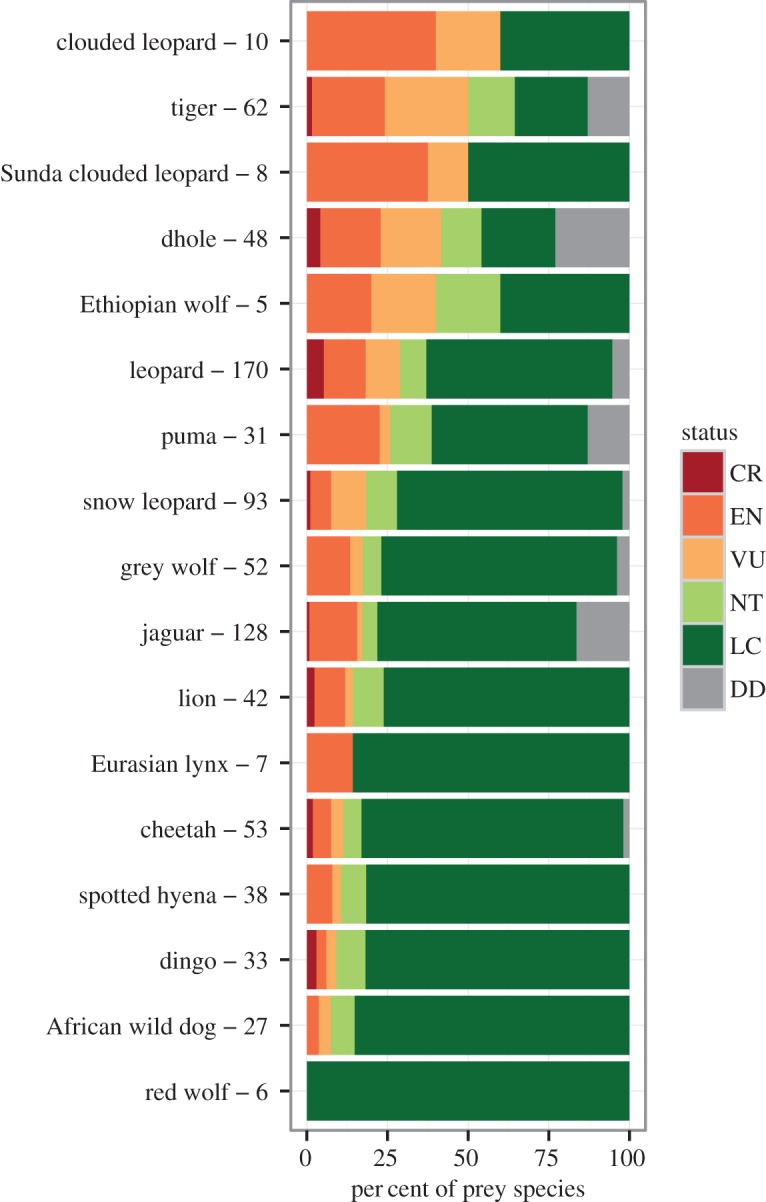
IUCN Red List conservation status of large carnivores' prey. Status categories are DD (data deficient), LC (least concern), NT (near threatened), VU (vulnerable), EN (endangered) and CR (critically endangered). Carnivores are ordered by decreasing percentage threatened (VU/EN/CR) prey from the top down. The numbers of prey species are shown after the large carnivore names.

**Figure 2.**
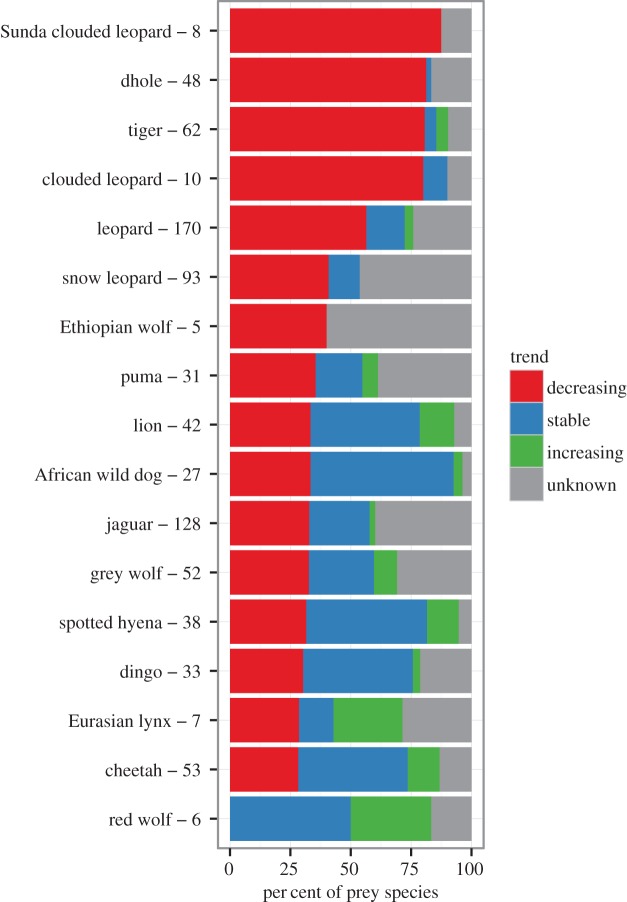
Population trends of large carnivores' prey. Carnivores are sorted by percentage of prey with decreasing population trends. The numbers of prey species are shown after the large carnivore names.

**Figure 3.**
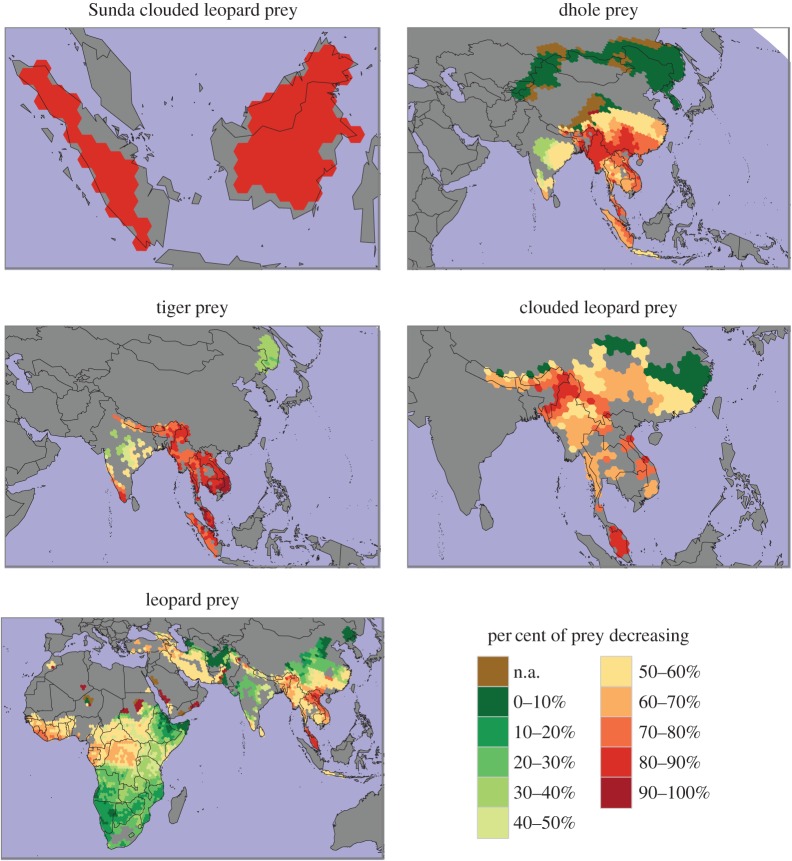
Maps showing the percentages of large carnivore prey species with decreasing population trends for the five large carnivores whose prey have the highest percentages of decreasing trends.

**Figure 4.**
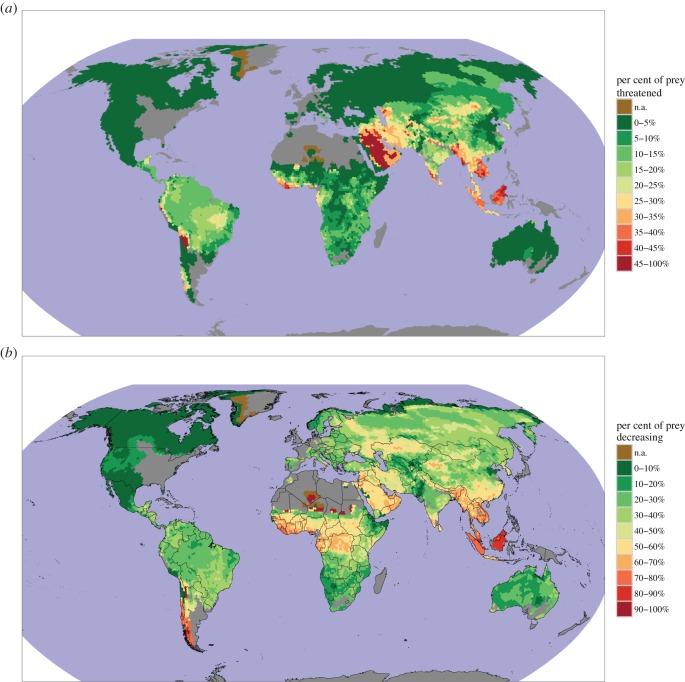
Percentages of all 494 prey species that are threatened (*a*) or have decreasing population trends (*b*).

The five large carnivores with the highest proportions of threatened prey were the clouded leopard (60%), Sunda clouded leopard (50%), tiger (50%), dhole (42%) and Ethiopian wolf (40%) (figure 1). All other carnivores had less than 30% of their prey threatened (figure 1; electronic supplementary material, figure S1). The five large carnivores with the highest proportions of prey with decreasing population trends were the Sunda clouded leopard (88%), tiger (81%), dhole (81%), clouded leopard (80%) and leopard (56%) (figures  2 and 3; electronic supplementary material, figure S2). Many prey species had unknown population trends. For example, 40% of the Ethiopian wolf's prey species have decreasing trends while the remaining 60% of its prey have unknown trends. At the continent level, prey endangerment rates were highest in Asia, South America and Africa, where large carnivores had an average of 33%, 22% and 18% of their prey threatened, respectively (figure 4; electronic supplementary material, figure S3). The corresponding percentages of prey with decreasing trends were 55%, 39% and 37% (figure 4; electronic supplementary material, figure S4).

The preferred prey lists contain 114 species, of which 20 (18%) were threatened (electronic supplementary material, table S2). Overall endangerment patterns for preferred prey species (electronic supplementary material, figures S5–S9) were similar to those for all prey species. The tiger and Ethiopian wolf have the highest rates of preferred prey endangerment—50% for both species (electronic supplementary material, figure S5). The clouded leopard and Sunda clouded leopard were excluded from the preferred prey analysis as their preferred prey species could not be determined from their diet study references.

Prey status declined from 1996 to 2008 for all but six of the large carnivores (table 2). No large carnivore had a net improvement in prey species status. Only one of the 494 prey species improved in status.

**Table 2.** Change in prey threat status. Total is the number of prey species LC–CR in 1996 and 2008. Declined/improved are the numbers of prey species that have declined/improved in status where LC < NT < VU < EN < CR. % Change is the net percentage change in prey endangerment.

species	total	declined	improved	% change
clouded leopard	10	3	0	−30.0
Sunda clouded leopard	8	2	0	−25.0
dhole	37	6	0	−16.2
tiger	54	6	1	−9.3
leopard	161	7	0	−4.3
puma	27	1	0	−3.7
snow leopard	91	3	0	−3.3
dingo	33	1	0	−3.0
jaguar	98	2	0	−2.0
gray wolf	50	1	0	−2.0
cheetah	52	1	0	−1.9
African wild dog	27	0	0	0
Ethiopian wolf	5	0	0	0
Eurasian lynx	7	0	0	0
lion	42	0	0	0
red wolf	6	0	0	0
spotted hyena	38	0	0	0

The primary threats faced by prey species were habitat change and hunting for meat, which threaten 19% and 16% of all prey species, respectively (electronic supplementary material, figure S10). The most common forms of habitat change threatening prey species were agriculture (12%) and deforestation (11%). Other common forms of hunting were hunting for body parts (10%), medicine (6%) and ornaments (5%).

**Figure 5.**
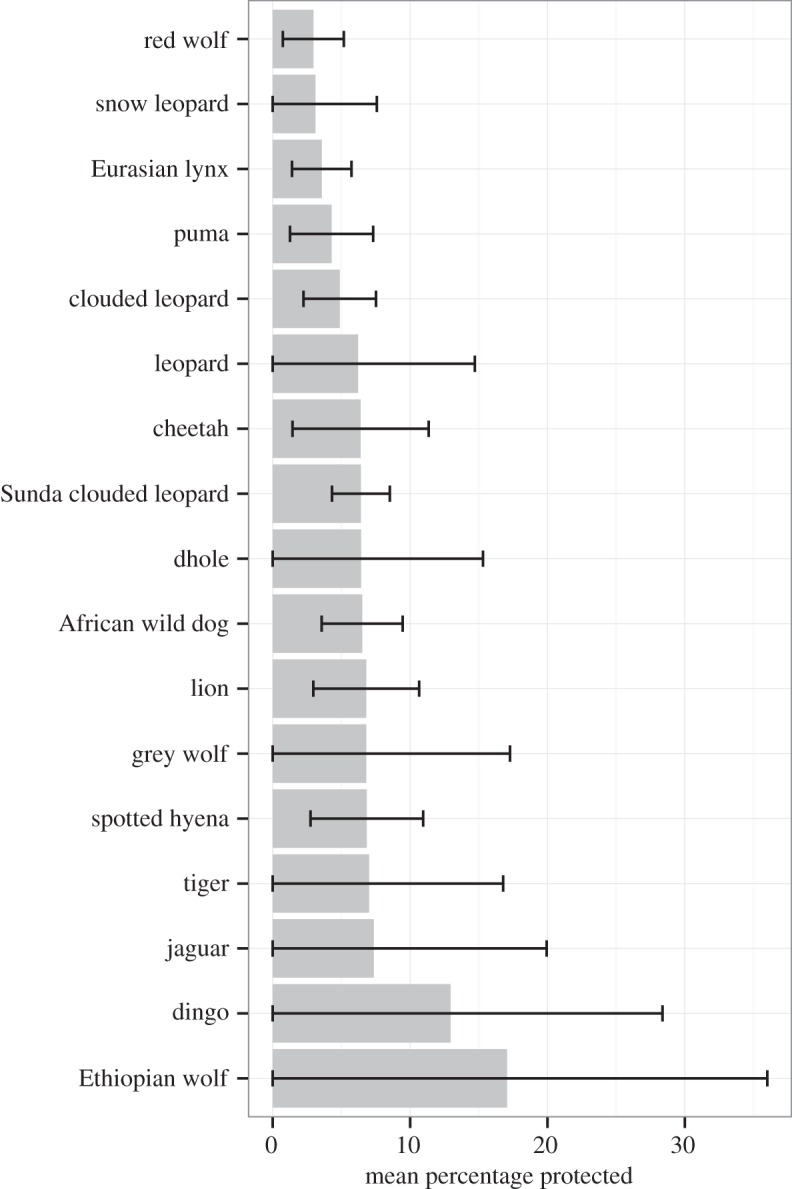
Mean percentages (with standard errors) of prey species ranges occurring inside protected areas. For example, the prey species of the dingo have an average of 13% of their ranges within protected areas.

The extent to which prey were protected varied widely, with the 494 total prey species having an average of 6.9% of their ranges protected (figure 5). The five large carnivores with the least protected prey were the red wolf (3%), snow leopard (3%), Eurasian lynx (4%), puma (4%) and clouded leopard (5%) (figure 5). Of these five species, the red wolf, snow leopard and clouded leopard were listed as threatened (table 1).

There is also wide variability in both prey species orders and masses (electronic supplementary material, figure S11). Of the 494 prey species identified, masses were available for 360 (73%). These 360 prey species cover a large range of masses: 0.01–0.1 kg (49 species), 0.1–1 kg (50), 1–10 kg (125), 10–100 kg (98), 100–1000 kg (33), 1000–10 000 kg (5). Altogether, the prey species span 16 different orders. The orders with at least five prey species were: even-toed ungulates (Cetartiodactyla), 133; opossums (Didelphimorphia), 88; primates (Primates), 87; rodents (Rodentia), 73; rabbits, hares and pika (Lagomorpha), 53; armadillos (Cingulata), 21; diprotodonts (Diprotodontia), 15; and odd-toed ungulates (Perissodactyla), 9.

Variability in prey consumed occurs both within and among large carnivore species. In terms of prey orders consumed, the four large carnivores with the greatest diet diversity were the leopard (seven orders), dingo (seven), cheetah (six) and jaguar (five). The carnivores that preyed on the fewest number of orders (2) were the African wild dog (*Lycaon pictus*), dhole, Ethiopian wolf and Eurasian lynx (electronic supplementary material, figure S11).

In total, we obtained 100 articles for our literature search (electronic supplementary material, table S3). Only four articles on the Sunda clouded leopard were found. Forty six of the articles mentioned prey endangerment status, prey depletion or the importance of prey to large carnivores (electronic supplementary material, figure S12). None of these 46 articles were about the Eurasian lynx, dingo, clouded leopard or Sunda clouded leopard (electronic supplementary material, figure S12).

## 4. Discussion

Our results show that prey endangerment leading to loss of prey base is a common threat faced by many large carnivores. In particular, the clouded leopard, Sunda clouded leopard, tiger, dhole and Ethiopian wolf all had highly threatened prey and were themselves threatened. With the exception of the Ethiopian wolf, all of these species have large portions of their ranges in Southeast Asia, where many prey species were endangered (figure 3). Prey in Southeast Asia face many threats related to high human population densities, hunting and habitat loss due to deforestation [32].

The continental results (electronic supplementary material, figures S3 and S4; see also figure 4) show that large carnivores' prey were more threatened in the developing world than in the developed world. This may be a consequence of many historic prey species (e.g. megaherbivores) having already been extirpated from Europe and the Americas due to overhunting and other factors [33]. Many of the remaining prey species have experienced major recoveries in the developed world to the point that some ungulate populations are now considered overabundant, especially in the absence of their native large predators [34]. The recovery of prey populations in Europe may have contributed to the recent large carnivore recoveries observed there, although other factors including increased public tolerance played a role as well [21]. Similarly, the higher rates of prey endangerment in the developing world, particularly Africa and Asia, have probably contributed to carnivores being more endangered there, with 9 of the 11 large carnivores endemic to these regions classified as threatened. In developing countries, carnivores may be endangered by both prey depletion and threats like habitat loss that are also faced by prey species.

The link between predator and prey endangerment provides additional rationale for conserving mammal prey species in the developing world. There is currently a funding mismatch with 32% of all threatened mammals found in the 40 most underfunded countries [35]. These 40 countries include Indonesia, Malaysia and many others where large carnivores are present [35]. Moreover, spending on terrestrial reserves in developing countries is less than 5% of what is needed for effective conservation [36]. As poverty rates in the developing world decline, conservation funding may increase and people could become less reliant on consuming wild meat for survival [37]. If this happens, prey populations could recover, and with supporting legislation and sufficient public tolerance, carnivores may also recover, paralleling the pattern of prey recoveries followed by predator recoveries seen in Europe.

Large carnivore diets typically include a wide range of prey (see electronic supplementary material, figure S11). While large carnivores are generally reliant on large prey, the majority of prey species were under 10 kg. This suggests that smaller prey species can be important food sources for many large carnivores and prey depletion analyses should consider abundances of both small and large prey. Seven large carnivores had median prey masses below 10 kg: jaguar (0.3 kg), Ethiopian wolf (0.4 kg), dingo (0.8 kg), red wolf (1.1 kg), snow leopard (1.5 kg), puma (4.2 kg) and clouded leopard (4.9 kg) (electronic supplementary material, figure S11). These species may be particularly reliant on small prey. In some cases, this could be a result of larger prey tending to be more endangered. Other carnivores may also switch to consuming smaller prey when larger prey are scarce, potentially decreasing their extinction risk. For example, African wild dogs can subsist on small prey such as Kirk's dikdiks (*Madoqua kirkii*) when abundant in regions where larger prey are depleted [38], and leopards may switch to consuming smaller prey when bushmeat hunting makes large prey scarce [19]. Low median prey masses may also be related to the inclusion of large numbers of small, rare prey in our original prey species lists. Restricting the analysis to preferred prey, just three carnivores had median preferred prey masses below 10 kg: Ethiopian wolf (0.1 kg), red wolf (1.4 kg) and jaguar (4.0 kg) (electronic supplementary material, figure S13).

Relatively low observed diet diversity could be due to lack of information on prey (likely with the clouded leopards), natural specialization (e.g. Ethiopian wolf and rodents) or due to declines in prey populations in the past (e.g. gray wolf and Eurasian lynx in Europe) or past and present (possibly dhole). In any case, carnivores with low diet breadth may be more vulnerable to prey depletion as it may be more difficult for them to switch to alternative prey when their preferred prey decline. In one study, rodents made up 88% of Ethiopian wolf diets by volume [39]. Ethiopian wolves are currently classified as critically endangered and their specialized diets consisting primarily of Afroalpine rodents may put them at greater risk of extinction. Although rodents are generally resilient species, the assessed rodent prey of Ethiopian wolves—the giant mole rat (*Tachyoryctes macrocephalus*) and black-clawed brush-furred rat (*Lophuromys melanonyx*)—are both threatened by livestock overgrazing of their habitat [8]. Even large carnivores with high observed diet breadth can be vulnerable to prey depletion. Anthropogenic threats to prey species such as habitat loss due to humans and hunting for meat often affect a wide array of prey species in contrast with more specialized threats like certain viruses (electronic supplementary material, figure S10).

While nearly half (46%) of the articles found in our literature search mentioned prey status or depletion, the majority of these articles were not focused on prey-related issues, but rather mentioned them briefly in passing, often when providing general conservation background information in the introduction section of the papers (electronic supplementary material, figure S12 and table S3). Our results exclude most articles on prey depletion as we only looked at the six most cited conservation articles for each carnivore. Several large carnivore species had specific conservation issues that dominated their search results. For example, the ecological effects of dingos, taxonomic issues associated with clouded leopards, and red wolves and hybridization.

### 4.1. Limitations

In our analysis, we have only considered terrestrial mammalian prey. The endangerment status of non-mammalian prey was not included in the results. However, a brief study of the predator-specific diet meta-analysis papers [40] suggests that non-mammals are generally not important food sources for large carnivores. We did not consider carnivores as potential prey for other carnivores as they are also seldom an important food source due to their naturally low abundances and other life-history traits [41]. Some of the prey species that were included in our analysis represent potential, rather than actual, prey. We attempted to assess the impact of excluding potential or uncommon prey by re-running key portions of our analysis on preferred prey only. Similarly, uncommon prey species may have been excluded from our analysis if they were not reported in our sources.

Prey depletion is a function of declining prey abundances, which are difficult to measure at a global scale. This analysis is reliant on the use of prey endangerment status as a proxy for prey abundance. Species can be classified as threatened due to declining or very low abundance, making this a reasonable proxy variable [24]. However, its use still represents a limitation of this analysis caused by the inability to measure abundances directly at this scale. Even accepting their use as a proxy variable, endangerment statuses also have the disadvantage that they are measured at the species level. This is a problem when endangerment varies across a species' range. For example, a prey species may be very endangered in one part of the world and overabundant in another part. Given the number and ranges of species involved in this analysis, there does not appear to be a more accurate data source that can be used. While our results should be interpreted carefully due to this limitation, it is likely that using global rather than local prey endangerment information underestimates the extent of prey depletion. If a prey species has several large, healthy populations, it is unlikely to be listed as threatened. On the other hand, it is common for prey species classified as ‘least concern’ or ‘near threatened’ to have threatened populations. This is particularly true in Africa where many ‘non-threatened’ ungulates like bushbuck (*Tragelaphus scriptus*) are locally threatened at certain sites due to bushmeat hunting [42].

In addition, this is a predator-centric project. Prey endangerment is viewed here as a potential driver of predator endangerment. However, prey endangerment is an important problem in and of itself as a large portion of mammals (both predators and prey) are currently threatened [43]. Moreover, overabundant predators may be a driver of prey endangerment—a possibility that was not explored in this project.

We assumed that predators' mammalian prey as reported in comprehensive diet meta-analyses [40] were representative of their historic prey. This may not be the case because historically preferred prey may now be too rare to appear in diet studies—a ‘shifting baseline’ problem [44]. For example, domestic animals recently made up an estimated 87% of the prey biomass of leopards in western Maharashtra, India [45]. In that study, the only wild prey found in leopard diets were rodents, small carnivores, primates and birds [45]. Assessing prey preferences in such cases is problematic as historic prey may have been extirpated from the study area. This issue is partially mitigated by our use of diet meta-analyses that probably include some studies from remote regions with relatively limited anthropogenic impacts.

### 4.2. Conservation implications

This analysis is the first of its kind and addresses an important conservation issue—depletion of prey as a threat to large carnivores—at a global scale. By analysing the status of prey for all the large carnivores as a group, we found significant evidence that the loss of prey species is a widespread issue and identified five large carnivore species (figure 3) that are particularly at risk. Of these five species, the leopard, clouded leopard and Sunda clouded leopard are ‘vulnerable’ and the tiger and dhole are ‘endangered’. While more work is needed to understand the extent to which these species are threatened due to prey depletion, our results indicate the importance of conserving prey to conserve large carnivores.

Recognition of the widespread occurrence of prey depletion motivates a holistic approach to conservation over purely predator-centric approaches. One of the ways this could be achieved is by strengthening management of protected areas which protect predators, prey and habitat. This is particularly important in Africa where many large carnivores reside and protected areas are often ineffective due to poaching, lack of funding and rapidly growing human populations [46]. In addition to strengthening management, enlarging and connecting existing reserves could help prevent isolation, reducing the impacts of harmful human-related edge effects [47]. As large carnivores range widely, are often persecuted by humans, and are highly dependent on prey availability, they can benefit greatly from expanded and strengthened protected area networks [47].

Large carnivores' broad diets mean that prey conservation efforts aimed at ensuring a robust prey base will tend to be most effective when they do not benefit individual prey species alone. For ungulates, a particularly important prey source for many carnivores (electronic supplementary material, figure S11), ‘collateral conservation’ (e.g. habitat protection, snare removal, certain programmes targeting other species) has been the primary factor preventing recent increases in endangerment [48]. More generally, large carnivores benefiting from prey conservation can also be viewed as a form of collateral conservation. Similarly, prey can benefit from conservation efforts aimed solely at their predators as predator populations often limit the abundances of competitively superior prey. While predator-centric conservation is certainly necessary (e.g. efforts to reduce hunting of threatened carnivores), ultimately, effective predator conservation cannot be accomplished without effective prey conservation [49].

## Supplementary Material

”carnivores prey paper - supplement”: Supporting figures and tables.

## Data accessibility

The datasets supporting this article have been uploaded as part of the electronic supplementary material.
