# Supplementary material for: Prey depletion as a threat to the world's large carnivores
Source: R Soc Open Sci. 2016 Aug 3;3(8):160252. doi: 10.1098/rsos.160252 (PMC5108949; doi:10.1098/rsos.160252)
Supplement: ”carnivores prey paper - supplement”: Supporting figures and tables. [file rsos160252supp1.docx]

**Supporting Information**

**Table S1.** Diet study sources for the large carnivores. Full references are listed below the table. Whenever possible, we referenced diet meta-analyses that synthesized diet studies throughout a carnivore’s range. Additional details are given in the methods section of the paper.

| **Common Name** | **Diet Reference(s)** |
| --- | --- |
| Cheetah | (1) |
| Gray wolf | (2) |
| Dingo | (3–8) |
| Red wolf | (9) |
| Ethiopian wolf | (10) |
| Spotted hyena | (11) |
| Dhole | (12) |
| African wild dog | (13) |
| Eurasian lynx | (14–16) |
| Sunda clouded leopard | (17–21) |
| Clouded leopard | (22,23) |
| Lion | (24) |
| Jaguar | (25) |
| Leopard | (26) |
| Tiger | (27) |
| Snow leopard | (28) |
| Puma | (29) |

**Diet references**

1. Hayward MW, Hofmeyr M, O’brien J, Kerley GIH. Prey preferences of the cheetah (Acinonyx jubatus)(Felidae: Carnivora): morphological limitations or the need to capture rapidly consumable prey before kleptoparasites arrive? J Zool. 2006;270(4):615–627.

2. Mech LD, Boitani L, editors. Wolves: Behavior, Ecology, and Conservation. University Of Chicago Press; 2007. 472 p.

3. Newsome AE, Corbett LK, Catling PC, Burt RJ. The Feeding Ecology of the Dingo. 1. Stomach Contents From Trapping in South-Eastern Australia, and the Non-Target Wildlife Also Caught in Dingo Traps. Wildl Res. 1983;10(3):477–486.

4. Newsome TM, Ballard G-A, Fleming PJS, van de Ven R, Story GL, Dickman CR. Human-resource subsidies alter the dietary preferences of a mammalian top predator. Oecologia. 2014 May;175(1):139–50.

5. Robertshaw JD, Harden RH. The Ecology of the Dingo in North-Eastern New South Wales. 2. Diet. Wildl Res. 1985;12(1):39–50.

6. Corbett LK, Newsome AE. The Feeding Ecology of the Dingo. III. Dietary Relationships with Widely Fluctuating Prey Populations in Arid Australia: An Hypothesis of Alternation of Predation. Oecologia. 1987 Jan 1;74(2):215–27.

7. Thomson PC. The behavioural ecology of dingoes in north-western Australia. III. Hunting and feeding behaviour, and diet. Wildl Res. 1992;19(5):531–541.

8. Allen BL, Leung LK-P. Assessing Predation Risk to Threatened Fauna from their Prevalence in Predator Scats: Dingoes and Rodents in Arid Australia. Hayward M, editor. PLoS ONE. 2012 May 1;7(5):e36426.

9. Dellinger J. Foraging and Spatial Ecology of Red Wolves (Canis rufus) in Northeastern North Carolina [Internet] [thesis]. 2011 [cited 2014 Aug 8]. Available from: https://etd.auburn.edu/handle/10415/2497

10. Sillero-Zubiri C, Gottelli D. Diet and Feeding Behavior of Ethiopian Wolves (Canis simensis). J Mammal. 1995 May 1;76(2):531–41.

11. Hayward MW. Prey preferences of the spotted hyaena (Crocuta crocuta) and degree of dietary overlap with the lion (Panthera leo). J Zool. 2006;270(4):606–614.

12. Selvan KM, Veeraswami GG, Hussain SA. Dietary preference of the Asiatic wild dog (Cuon alpinus). Mamm Biol - Z Für Säugetierkd. 2013 Nov;78(6):486–9.

13. Hayward MW, O’Brien J, Hofmeyr M, Kerley GI. Prey preferences of the African wild dog Lycaon pictus (Canidae: Carnivora): ecological requirements for conservation. J Mammal. 2006;87(6):1122–1131.

14. Nowicki P. Food habits and diet of the lynx (Lynx lynx) in Europe. J Wildl Res. 1997;2(2):161–166.

15. Herfindal I, Linnell JDC, Odden J, Nilsen EB, Andersen R. Prey density, environmental productivity and home-range size in the Eurasian lynx (Lynx lynx). J Zool. 2005 Jan;265(1):63–71.

16. Krofel M, Huber D, Kos I. Diet of Eurasian lynx Lynx lynx in the northern Dinaric Mountains (Slovenia and Croatia): Importance of edible dormouse Glis glis as alternative prey. Acta Theriol (Warsz). 2011 Oct;56(4):315–22.

17. Rabinowitz A, Andau P, Chai PP. The clouded leopard in Malaysian Borneo. Oryx. 1987;21(2):107–111.

18. Gordon CH, Stewart A-ME, Meijaard E. Correspondence regarding “Clouded leopards, the secretive top-carnivore of South-East Asian rainforests: their distribution, status and conservation needs in Sabah, Malaysia.” BMC Ecol. 2007;7(1):5.

19. Matsuda I, Tuuga A, Higashi S. Clouded leopard (Neofelis diardi) predation on proboscis monkeys (Nasalis larvatus) in Sabah, Malaysia. Primates. 2008 Jul;49(3):227–31.

20. Mohamed A, Samejima H, Wilting A. Records of five Bornean cat species from Deramakot Forest Reserve in Sabah, Malaysia. Cat News. 2009;51:12–15.

21. Ross J, Hearn AJ, Johnson PJ, Macdonald DW. Activity patterns and temporal avoidance by prey in response to Sunda clouded leopard predation risk: Activity of Sunda clouded leopards and their prey. J Zool. 2013 Jun;290(2):96–106.

22. Grassman Jr LI, Tewes ME, Silvy NJ, Kreetiyutanont K. Ecology of three sympatric felids in a mixed evergreen forest in north-central Thailand. J Mammal. 2005;86(1):29–38.

23. Ngoprasert D, Lynam AJ, Sukmasuang R, Tantipisanuh N, Chutipong W, Steinmetz R, et al. Occurrence of Three Felids across a Network of Protected Areas in Thailand: Prey, Intraguild, and Habitat Associations. Biotropica. 2012 Nov;44(6):810–7.

24. Hayward MW, Kerley GIH. Prey preferences of the lion (Panthera leo). J Zool. 2005 Nov 1;267(3):309–22.

25. Seymour KL. Panthera onca. Mamm Species. 1989 Oct 26;(340):1–9.

26. Hayward MW, Henschel P, O’brien J, Hofmeyr M, Balme G, Kerley GIH. Prey preferences of the leopard (Panthera pardus). J Zool. 2006;270(2):298–313.

27. Hayward MW, Jędrzejewski W, Jêdrzejewska B. Prey preferences of the tiger Panthera tigris. J Zool. 2012 Mar 1;286(3):221–31.

28. Lyngdoh S, Shrotriya S, Goyal SP, Clements H, Hayward MW, Habib B. Prey Preferences of the Snow Leopard (Panthera uncia): Regional Diet Specificity Holds Global Significance for Conservation. PLoS ONE. 2014 Feb 12;9(2):e88349.

29. Iriarte JA, Franklin WL, Johnson WE, Redford KH. Biogeographic variation of food habits and body size of the America puma. Oecologia. 1990;85(2):185–190.

**Table S2.** Information on the 494 prey species in our analysis. “Predators” lists the predators of each prey species (using the first 2 letters of each word of the predator’s common name), “RLS” and “Trend” are the IUCN Red List status (2008 or later) and population trend respectively, “Mass” is the mass in kg, and “% in PAs” is the percentage of the prey’s range within protected areas. Predators for which a prey species is preferred are marked with an asterisk.

| **Prey species** | **Common Name** | **Predators** | **RLS** | **Trend** | **Mass** | **% in PAs** |
| --- | --- | --- | --- | --- | --- | --- |
| **Cetartiodactyla (even-toed ungulates)** | | | | | | |
| Elaphodus cephalophus | Tufted deer | GrWo* | NT | Dec | 23.09 | 0% |
| Muntiacus crinifrons | Black muntjac | Dh*,Le | VU | Dec | 18.59 | 0% |
| Moschus berezovskii | Chinese forest musk deer | SnLe,Ti | EN | Dec | 12.48 | 0% |
| Cephalophus adersi | Aders' duiker | Le | CR | Dec | 9.25 | 0% |
| Bos mutus | Wild yak | SnLe | VU | Dec |  | 0% |
| Muntiacus puhoatensis | Puhoat muntjac | Dh*,Le,Ti | DD | Unk |  | 0% |
| Tragulus versicolor | Silver-backed chevrotain | Dh | DD | Dec |  | 0% |
| Tragulus williamsoni | Williamson's chevrotain | Dh | DD | Dec |  | 0% |
| Connochaetes gnou | Black wildebeest | Ch,SpHy | LC | Inc | 156.55 | 0% |
| Damaliscus pygargus | Blesbok | AfWiDo,Ch*,Le | LC | Stable | 77.79 | 0% |
| Hydropotes inermis | Chinese water deer | GrWo* | VU | Dec | 12.76 | 0% |
| Raphicerus melanotis | Cape grysbok | Ch | LC | Stable | 10.50 | 0% |
| Muntiacus reevesi | Chinese muntjak | Dh*,GrWo*,Le | LC | Dec | 13.50 | 0% |
| Boselaphus tragocamelus | Nilgai | Le,Ti | LC | Stable | 182.25 | 0% |
| Tetracerus quadricornis | Chousingha | Ti | VU | Dec | 19.28 | 1% |
| Mazama pandora | Yucatan brown brocket | Ja | VU | Dec |  | 1% |
| Gazella bennettii | Chinkara | Ti | LC | Stable | 18.92 | 1% |
| Moschiola indica | Indian chevrotain | Dh | LC | Unk |  | 1% |
| Pseudois nayaur | Bharal | SnLe* | LC | Unk | 52.34 | 1% |
| Muntiacus rooseveltorum | Roosevelts' barking deer | Dh*,Le,Ti | DD | Dec | 10.76 | 1% |
| Mazama gouazoubira | Brown brocket | Ja | LC | Dec | 16.63 | 1% |
| Capra falconeri | Markhor | SnLe | EN | Dec | 54.30 | 1% |
| Moschus chrysogaster | Alpine musk deer | SnLe,Ti | EN | Dec | 13.70 | 1% |
| Ovis orientalis | Cyprian wild sheep | EuLy*,GrWo*,SnLe | VU | Dec |  | 1% |
| Mazama nana | Brazilian dwarf brocket | Ja | DD | Unk | 16.50 | 1% |
| Axis axis | Axis deer | Dh*,GrWo*,Le,Li,Ti | LC | Unk | 69.50 | 1% |
| Muntiacus truongsonensis | Annam black muntjac | Dh*,Le,Ti | DD | Dec |  | 1% |
| Dama dama | Fallow deer | GrWo* | LC | Unk | 57.23 | 2% |
| Gazella subgutturosa | Goitered gazelle | SnLe | VU | Dec | 26.98 | 2% |
| Capreolus pygargus | Eastern roe deer | GrWo* | LC | Dec | 41.37 | 2% |
| Sus scrofa | Eurasian wild pig | ClLe,Dh*,GrWo*,Le,SnLe,Ti* | LC | Unk | 84.47 | 2% |
| Axis porcinus | Hog deer | ClLe,GrWo*,Ti | EN | Dec | 37.45 | 2% |
| Moschus cupreus | Kashmir muskdeer | SnLe | EN | Dec |  | 2% |
| Capreolus capreolus | European roe deer | EuLy*,GrWo* | LC | Inc | 22.50 | 2% |
| Muntiacus vaginalis | Barking deer | Dh*,GrWo*,Le,Ti | LC | Dec |  | 2% |
| Tragulus javanicus | Java mousedeer | Dh | DD | Unk | 1.89 | 3% |
| Philantomba maxwellii | Maxwell's duiker | Le | LC | Dec | 8.56 | 3% |
| Catagonus wagneri | Chacoan peccary | Pu | EN | Dec | 35.57 | 3% |
| Kobus megaceros | Nile lechwe | Ch | EN | Dec | 85.81 | 3% |
| Cephalophus ogilbyi | Ogilby's duiker | Le | LC | Dec | 18.39 | 3% |
| Cephalophus natalensis | Natal duiker | Le,SpHy | LC | Dec | 12.73 | 3% |
| Cephalophus niger | Black duiker | Le | LC | Dec | 19.09 | 3% |
| Muntiacus putaoensis | Leaf deer | Dh*,GrWo*,Le,Ti | DD | Dec |  | 3% |
| Ovis ammon | Argali | SnLe | NT | Dec | 114.00 | 3% |
| Cervus elaphus | Bactrian deer | EuLy*,GrWo*,Ti* | LC | Inc | 240.87 | 3% |
| Rusa unicolor | Sambar | ClLe,Dh*,GrWo*,Le,Li,SuClLe,Ti* | VU | Dec | 177.52 | 3% |
| Redunca fulvorufula | Mountain reedbuck | AfWiDo,Ch,Le,Li,SpHy | LC | Stable | 29.35 | 3% |
| Tragelaphus angasii | Nyala | AfWiDo,Ch,Le,Li,SpHy | LC | Stable | 87.62 | 4% |
| Cephalophus nigrifrons | Black-fronted duiker | Le | LC | Dec | 14.68 | 4% |
| Moschus moschiferus | Siberian musk deer | SnLe,Ti | VU | Dec | 13.32 | 4% |
| Cephalophus dorsalis | Bay duiker | Le | LC | Dec | 20.00 | 4% |
| Cephalophus zebra | Banded duiker | Le | VU | Dec | 15.66 | 4% |
| Alces alces | Elk | GrWo* | LC | Inc | 461.90 | 4% |
| Cephalophus silvicultor | Yellow-backed duiker | Le | LC | Dec | 62.01 | 4% |
| Tragelaphus imberbis | Lesser kudu | AfWiDo*,Ch,Le,Li,SpHy | NT | Dec | 94.32 | 4% |
| Blastocerus dichotomus | Marsh deer | Ja | VU | Dec | 112.52 | 4% |
| Tragelaphus scriptus | Bushbuck | AfWiDo*,Ch,Le*,Li,SpHy | LC | Stable | 43.25 | 4% |
| Odocoileus virginianus | Key deer | GrWo*,Pu*,ReWo* | LC | Stable | 75.90 | 4% |
| Cephalophus harveyi | Harvey's duiker | Le | LC | Dec |  | 4% |
| Philantomba monticola | Blue duiker | Ch,Le | LC | Stable | 4.90 | 5% |
| Redunca arundinum | Common reedbuck | AfWiDo,Ch,Le,Li,SpHy | LC | Stable | 58.06 | 5% |
| Rucervus duvaucelii | Barasingha | GrWo*,Ti* | VU | Dec | 171.22 | 5% |
| Cephalophus callipygus | Peter's duiker | Le | LC | Dec | 19.08 | 5% |
| Mazama temama | Central american red brocket | Ja | DD | Dec |  | 5% |
| Oreotragus oreotragus | Klipspringer | AfWiDo,Ch,Le,Li,SpHy | LC | Stable | 13.49 | 5% |
| Cephalophus jentinki | Jentink's duiker | Le | EN | Dec | 68.49 | 5% |
| Cephalophus rufilatus | Red-flanked duiker | Le | LC | Dec | 12.12 | 5% |
| Pudu mephistophiles | Northern pudu | Pu | VU | Dec | 9.60 | 5% |
| Sylvicapra grimmia | Common duiker | AfWiDo,Ch,Le*,Li,SpHy | LC | Stable | 15.64 | 5% |
| Redunca redunca | Bohar reedbuck | AfWiDo,Ch,Le,Li,SpHy | LC | Dec | 43.29 | 5% |
| Hyemoschus aquaticus | Water chevrotain | Le | LC | Dec | 10.85 | 5% |
| Ourebia ourebi | Oribi | AfWiDo,Ch,Le,Li | LC | Dec | 17.19 | 5% |
| Cervus nippon | Shansi sika | GrWo*,Ti | LC | Inc | 53.00 | 5% |
| Tragelaphus spekii | Marshbuck | Le | LC | Dec | 75.55 | 5% |
| Bos gaurus | Gaur | Dh,Ti | VU | Dec |  | 5% |
| Kobus ellipsiprymnus | Waterbuck | AfWiDo,Ch,Le,Li,SpHy | LC | Dec | 204.39 | 5% |
| Cephalophus leucogaster | White-bellied duiker | Le | LC | Dec | 13.21 | 5% |
| Phacochoerus africanus | Common warthog | AfWiDo,Ch,Le,Li,SpHy | LC | Stable | 82.50 | 5% |
| Pecari tajacu | Collared peccary | Ja*,Pu | LC | Stable | 21.13 | 6% |
| Potamochoerus larvatus | Bushpig | Ch,Le,Li,SpHy | LC | Stable | 69.06 | 6% |
| Tragelaphus strepsiceros | Greater kudu | AfWiDo*,Ch,Le,Li,SpHy | LC | Stable | 206.06 | 6% |
| Muntiacus vuquangensis | Giant muntjac | Dh*,Le,Ti | EN | Dec | 36.69 | 6% |
| Tragelaphus eurycerus | Bongo | Le | NT | Dec | 271.00 | 6% |
| Raphicerus sharpei | Sharpe's grysbok | AfWiDo,Ch,Le,Li,SpHy | LC | Stable | 9.40 | 6% |
| Cephalophus weynsi | Weyn's duiker | Le | LC | Dec |  | 6% |
| Muntiacus muntjak | Barking deer | ClLe,Dh*,Le,SuClLe,Ti | LC | Dec | 17.61 | 6% |
| Tragelaphus oryx | Common eland | AfWiDo,Ch,Le,Li,SpHy | LC | Stable |  | 6% |
| Rangifer tarandus | Caribou | EuLy*,GrWo* | LC | Stable | 109.09 | 6% |
| Alces americanus | Moose | GrWo* | LC | Stable | 541.46 | 7% |
| Rupicapra rupicapra | Alpine chamois | EuLy*,GrWo* | LC | Unk | 33.27 | 7% |
| Kobus kob | Kob | Li | LC | Dec | 80.04 | 7% |
| Odocoileus hemionus | Black-tailed deer | GrWo*,Pu* | LC | Stable | 84.56 | 7% |
| Mazama americana | Red brocket | Ja | DD | Unk | 20.55 | 7% |
| Syncerus caffer | African buffalo | AfWiDo,Ch,Le,Li*,SpHy | LC | Dec | 592.67 | 7% |
| Tayassu pecari | White-lipped peccary | Ja*,Pu | VU | Dec | 31.80 | 7% |
| Aepyceros melampus | Black-faced impala | AfWiDo*,Ch*,Le*,Li,SpHy | LC | Stable | 52.59 | 7% |
| Nanger granti | Grant's gazelle | AfWiDo,Ch*,Le,Li,SpHy | LC | Dec | 55.46 | 7% |
| Moschus fuscus | Black musk deer | SnLe,Ti | EN | Dec | 13.70 | 7% |
| Alcelaphus buselaphus | Hartebeest | AfWiDo,Ch,Le,Li,SpHy | LC | Dec | 160.94 | 7% |
| Hippotragus equinus | Roan antelope | Ch,Le,Li,SpHy | LC | Dec | 264.17 | 7% |
| Raphicerus campestris | Steenbok | AfWiDo,Ch,Le,Li,SpHy | LC | Stable | 11.66 | 7% |
| Muntiacus gongshanensis | Gongshan muntjac | Dh*,GrWo*,Le,Ti | DD | Dec | 18.59 | 8% |
| Naemorhedus goral | Goral | SnLe,Ti | NT | Dec | 28.80 | 8% |
| Mazama rufina | Dwarf red brocket | Ja | VU | Dec | 21.10 | 8% |
| Tragulus napu | Balabac chevrotain | Dh,SuClLe | LC | Dec | 5.27 | 8% |
| Neotragus batesi | Bates' pygmy antelope | Le | LC | Stable | 2.97 | 8% |
| Sus barbatus | Bearded pig | SuClLe | VU | Dec | 135.81 | 8% |
| Mazama bororo | Small red brocket | Ja | VU | Dec |  | 8% |
| Capra sibirica | Asiatic ibex | SnLe* | LC | Unk | 130.00 | 8% |
| Tragulus kanchil | Lesser malay chevrotain | Dh,SuClLe | LC | Unk |  | 8% |
| Mazama nemorivaga | Amazonian brown brocket | Ja | LC | Dec |  | 9% |
| Connochaetes taurinus | Blue & white-bearded wildebeest | AfWiDo,Ch,Le,Li*,SpHy | LC | Stable | 198.62 | 9% |
| Hemitragus jemlahicus | Himalayan tahr | SnLe | NT | Dec | 68.62 | 10% |
| Antidorcas marsupialis | Springbok | AfWiDo,Ch*,Le,Li,SpHy | LC | Inc | 33.57 | 10% |
| Moschus leucogaster | Himalayan muskdeer | SnLe,Ti | EN | Dec |  | 10% |
| Hippopotamus amphibius | Common hippopotamus | Ch,Le,Li,SpHy | VU | Dec | 1536.31 | 11% |
| Damaliscus lunatus | Tiang | AfWiDo,Ch,Le,Li,SpHy | LC | Dec | 136.00 | 11% |
| Oryx gazella | Gemsbok | Ch,Le,Li*,SpHy* | LC | Stable | 188.40 | 12% |
| Pudu puda | Chilean pudu | Pu | VU | Dec | 9.64 | 13% |
| Eudorcas thomsonii | Thomson's gazelle | AfWiDo*,Ch*,Le,Li,SpHy | NT | Dec | 22.91 | 14% |
| Giraffa camelopardalis | Giraffe | Ch,Le,Li*,SpHy | LC | Dec | 964.66 | 14% |
| Bos javanicus | Banteng | Ti | EN | Dec | 635.97 | 16% |
| Ovis canadensis | Bighorn sheep | GrWo* | LC | Stable | 74.65 | 17% |
| Mazama chunyi | Chunyi | Ja | VU | Dec | 16.07 | 18% |
| Muntiacus montanus | Sumatran mountain muntjac | Dh*,Ti | DD | Unk |  | 20% |
| Mazama bricenii | Mérida brocket | Ja | VU | Dec | 16.50 | 23% |
| Ovibos moschatus | Muskox | GrWo* | LC | Stable | 312.50 | 25% |
| Kobus leche | Southern lechwe | Ch | LC | Stable | 88.65 | 28% |
| Oreamnos americanus | Mountain goat | GrWo* | LC | Stable | 72.11 | 30% |
| Muntiacus feae | Fea's muntjac | Dh*,Le,Ti | DD | Unk | 22.00 | 40% |
| Bison bison | American bison | GrWo* | NT | Stable | 624.58 | 45% |
| Dama mesopotamica | Mesopotamian fallow deer | GrWo* | EN | Inc |  | 49% |
| Cephalophus spadix | Abbott's duiker | Le | EN | Dec | 56.86 | 59% |
| **Chiroptera (bats)** | | | | | | |
| Nyctophilus geoffroyi | Lesser long-eared bat | Di | LC | Stable |  | 7% |
| **Cingulata (armadillos)** | | | | | | |
| Dasypus hybridus | Southern long-nosed armadillo | Ja*,Pu | NT | Dec | 1.50 | 0% |
| Chaetophractus vellerosus | Screaming hairy armadillo | Ja*,Pu | LC | Stable | 0.93 | 1% |
| Tolypeutes matacus | Southern three-banded armadillo | Ja*,Pu | NT | Dec | 1.30 | 1% |
| Cabassous tatouay | Greater naked-tailed armadillo | Ja*,Pu | LC | Unk | 5.35 | 1% |
| Calyptophractus retusus | Burmeister's armadillo | Ja*,Pu | DD | Unk | 0.13 | 1% |
| Chaetophractus nationi | Andean hairy armadillo | Ja*,Pu | VU | Dec | 2.11 | 1% |
| Chlamyphorus truncatus | Lesser fairy armadillo | Pu | DD | Unk | 0.08 | 1% |
| Chaetophractus villosus | Large hairy armadillo | Ja*,Pu | LC | Inc | 4.37 | 1% |
| Cabassous chacoensis | Chacoan naked-tailed armadillo | Ja*,Pu | NT | Unk | 1.49 | 2% |
| Zaedyus pichiy | Pichi | Pu | NT | Dec | 1.45 | 2% |
| Dasypus septemcinctus | Brazilian lesser long-nosed armadillo | Ja*,Pu | LC | Unk | 1.53 | 2% |
| Euphractus sexcinctus | Six-banded armadillo | Ja*,Pu | LC | Stable | 4.73 | 3% |
| Dasypus yepesi | Yepes’s mulita | Ja*,Pu | DD | Unk |  | 3% |
| Tolypeutes tricinctus | Brazilian three-banded armadillo | Ja*,Pu | VU | Dec | 1.47 | 3% |
| Dasypus sabanicola | Llanos long-nosed armadillo | Ja*,Pu | LC | Unk | 1.15 | 4% |
| Dasypus novemcinctus | Common long-nosed armadillo | Ja*,Pu | LC | Inc | 3.95 | 5% |
| Dasypus pilosus | Hairy long-nosed armadillo | Ja*,Pu | VU | Unk | 4.45 | 6% |
| Cabassous centralis | Northern naked-tailed armadillo | Ja*,Pu | DD | Unk | 3.67 | 7% |
| Cabassous unicinctus | Southern naked-tailed armadillo | Ja*,Pu | LC | Unk | 3.99 | 7% |
| Priodontes maximus | Giant armadillo | Ja*,Pu | VU | Dec | 40.64 | 7% |
| Dasypus kappleri | Greater long-nosed armadillo | Ja*,Pu | LC | Unk | 9.70 | 10% |
| **Dasyuromorphia (quolls and dunnarts)** | | | | | | |
| Sminthopsis macroura | Stripe-faced dunnart | Di | LC | Unk | 0.02 | 4% |
| Sminthopsis crassicaudata | Fat-tailed dunnart | Di | LC | Stable | 0.02 | 6% |
| Antechinus stuartii | Brown antechinus | Di | LC | Stable | 0.03 | 20% |
| Antechinus swainsonii | Dusky antechinus | Di | LC | Unk | 0.06 | 20% |
| **Didelphimorphia (opossums)** | | | | | | |
| Marmosops handleyi | Handley's slender mouse opossum | Ja | CR | Dec | 0.03 | 0% |
| Marmosops cracens | Slim-faced slender mouse opossum | Ja | DD | Unk | 0.03 | 0% |
| Cryptonanus unduaviensis |  | Ja | DD | Unk |  | 0% |
| Marmosops creightoni |  | Ja | DD | Unk |  | 0% |
| Marmosops ocellatus |  | Ja | LC | Stable |  | 0% |
| Monodelphis handleyi | Handley's short-tailed opossum | Ja | NT | Unk |  | 0% |
| Monodelphis maraxina | Marajó short-tailed opossum | Ja | DD | Dec |  | 0% |
| Philander olrogi | Olrog`s four-eyed opossum | Ja | DD | Unk |  | 0% |
| Tlacuatzin canescens | Grayish mouse opossum | Ja | LC | Stable | 0.05 | 0% |
| Chacodelphys formosa |  | Ja | VU | Dec |  | 0% |
| Thylamys macrurus | Long-tailed fat-tailed opossum | Ja | NT | Dec | 0.03 | 0% |
| Thylamys pulchellus |  | Ja | LC | Unk |  | 0% |
| Monodelphis rubida | Chestnut-striped opossum | Ja | DD | Dec | 0.05 | 0% |
| Thylamys pusillus | Small fat-tailed opossum | Ja | LC | Dec | 0.03 | 0% |
| Thylamys venustus | Buff-bellied fat-tailed mouse opossum | Ja | DD | Unk |  | 1% |
| Monodelphis americana | Northern three-striped opossum | Ja | LC | Dec | 0.02 | 1% |
| Monodelphis dimidiata | Eastern short-tailed opossum | Ja | LC | Stable | 0.05 | 1% |
| Monodelphis kunsi | Pygmy short-tailed opossum | Ja | LC | Stable | 0.01 | 1% |
| Thylamys velutinus | Dwarf fat-tailed mouse opossum | Ja | LC | Unk | 0.02 | 1% |
| Thylamys sponsorius |  | Ja | LC | Unk |  | 1% |
| Monodelphis theresa | Southern three-striped opossum | Ja | DD | Dec | 0.11 | 1% |
| Didelphis virginiana | Virginia opossum | Ja | LC | Inc | 2.44 | 1% |
| Cryptonanus chacoensis | Chacoan mouse opossum | Ja | LC | Stable |  | 1% |
| Cryptonanus agricolai | Agricola's gracile opossum | Ja | DD | Unk |  | 1% |
| Didelphis albiventris | White-eared opossum | Ja | LC | Stable | 1.03 | 1% |
| Didelphis aurita | Big-eared opossum | Ja | LC | Stable | 1.11 | 2% |
| Marmosa constantiae | Bay-colored mouse opossum | Ja | LC | Unk |  | 2% |
| Philander frenatus | Southeastern four-eyed opossum | Ja | LC | Unk |  | 2% |
| Monodelphis domestica | Gray short-tailed opossum | Ja | LC | Stable | 0.09 | 2% |
| Marmosa paraguayanus | Tate's woolly mouse opossum | Ja | LC | Stable |  | 2% |
| Gracilinanus microtarsus | Brazilian gracile mouse opossum | Ja | LC | Unk | 0.03 | 2% |
| Marmosops incanus | Gray slender mouse opossum | Ja | LC | Unk | 0.06 | 2% |
| Monodelphis scalops | Long-nosed short-tailed opossum | Ja | LC | Dec | 0.76 | 2% |
| Monodelphis iheringi | Ihering's short-tailed opossum | Ja | DD | Dec | 0.11 | 2% |
| Thylamys cinderella | Cinderella fat-tailed mouse opossum | Ja | LC | Unk |  | 2% |
| Lutreolina crassicaudata | Little water opossum | Ja | LC | Unk | 0.56 | 3% |
| Thylamys karimii | Karimi's fat-tailed mouse opossum | Ja | VU | Dec |  | 3% |
| Marmosa xerophila | Dryland mouse opossum | Ja | VU | Dec | 0.05 | 3% |
| Gracilinanus agilis | Agile gracile mouse opossum | Ja | LC | Unk | 0.02 | 3% |
| Marmosa mexicana | Mexican mouse opossum | Ja | LC | Stable | 0.05 | 4% |
| Monodelphis emiliae | Emilia's short-tailed opossum | Ja | LC | Unk | 0.05 | 4% |
| Philander mcilhennyi | Mcilhenny's four-eyed opossum | Ja | LC | Unk |  | 4% |
| Didelphis pernigra |  | Ja | LC | Stable |  | 5% |
| Gracilinanus marica | Northern gracile mouse opossum | Ja | LC | Dec | 0.02 | 5% |
| Marmosops bishopi |  | Ja | LC | Unk |  | 5% |
| Caluromys philander | Bare-tailed woolly opossum | Ja | LC | Dec | 0.25 | 5% |
| Caluromys derbianus | Central american woolly opossum | Ja | LC | Dec | 0.33 | 5% |
| Philander mondolfii | Mondolfi's four-eyed opossum | Ja | LC | Unk |  | 5% |
| Gracilinanus emiliae | Emilia's gracile mouse opossum | Ja | DD | Unk | 0.01 | 6% |
| Marmosops paulensis | Brazilian slender opossum | Ja | LC | Unk | 0.04 | 6% |
| Gracilinanus aceramarcae | Aceramarca gracile mouse opossum | Ja | LC | Unk | 0.02 | 6% |
| Marmosa robinsoni | Robinson's mouse opossum | Ja | LC | Stable | 0.06 | 6% |
| Glironia venusta | Bushy-tailed opossum | Ja | LC | Unk | 0.11 | 6% |
| Caluromys lanatus | Brown-eared wooly opossum | Ja | LC | Dec | 0.35 | 7% |
| Marmosops juninensis |  | Ja | VU | Dec |  | 7% |
| Chironectes minimus | Water opossum | Ja | LC | Dec | 0.97 | 7% |
| Marmosops noctivagus | White-bellied slender mouse opossum | Ja | LC | Stable | 0.04 | 7% |
| Marmosa regina | Short-furred woolly mouse opossum | Ja | LC | Stable |  | 7% |
| Metachirus nudicaudatus | Brown four-eyed opossum | Ja | LC | Stable | 0.36 | 7% |
| Marmosa rubra | Red mouse opossum | Ja | DD | Unk | 0.06 | 7% |
| Marmosops fuscatus | Gray-bellied slender mouse opossum | Ja | DD | Unk | 0.05 | 7% |
| Monodelphis adusta | Sepia short-tailed opossum | Ja | LC | Stable | 0.04 | 8% |
| Marmosa demerarae | Long-furred woolly mouse opossum | Ja | LC | Stable |  | 8% |
| Didelphis marsupialis | Black-eared opossum | Ja | LC | Stable | 1.14 | 8% |
| Monodelphis palliolata | Hooded red-sided opossum | Ja | LC | Unk |  | 8% |
| Marmosops neblina |  | Ja | LC | Stable |  | 9% |
| Marmosops impavidus | Andean slender mouse opossum | Ja | LC | Stable | 0.04 | 9% |
| Marmosa murina | Linnaeus's mouse opossum | Ja | LC | Stable | 0.04 | 9% |
| Philander opossum | Gray four-eyed opossum | Ja | LC | Stable | 0.43 | 9% |
| Philander deltae | Deltaic four-eyed opossum | Ja | LC | Unk |  | 10% |
| Monodelphis glirina | Amazonian red-sided opossum | Ja | LC | Unk |  | 10% |
| Hyladelphys kalinowskii | Kalinowski's mouse opossum | Ja | LC | Unk |  | 11% |
| Marmosa lepida | Little rufous mouse opossum | Ja | LC | Stable | 0.01 | 11% |
| Monodelphis osgoodi | Osgood's short-tailed opossum | Ja | LC | Unk | 0.11 | 12% |
| Marmosa alstoni | Alston's woolly mouse opossum | Ja | LC | Stable |  | 12% |
| Marmosops invictus | Slaty slender mouse opossum | Ja | LC | Stable | 0.03 | 12% |
| Marmosops parvidens | Delicate slender mouse opossum | Ja | LC | Unk | 0.02 | 13% |
| Philander andersoni | Anderson's four-eyed opossum | Ja | LC | Stable | 0.33 | 13% |
| Gracilinanus dryas | Wood sprite gracile mouse opossum | Ja | NT | Dec | 0.02 | 14% |
| Marmosops pinheiroi | Pinheiro's slender opossum | Ja | LC | Unk |  | 14% |
| Monodelphis brevicaudata | Northern red-sided opossum | Ja | LC | Unk | 0.08 | 16% |
| Marmosa quichua | Quechuan mouse opossum | Ja | LC | Dec |  | 20% |
| Caluromysiops irrupta | Black-shouldered opossum | Ja | LC | Dec | 0.26 | 21% |
| Didelphis imperfecta | Guianan white-eared opossum | Ja | LC | Stable |  | 22% |
| Marmosa andersoni | Anderson's mouse opossum | Ja | DD | Unk | 0.05 | 43% |
| Monodelphis reigi | Reig's opossum | Ja | VU | Unk |  | 48% |
| Marmosa tyleriana | Tyler's mouse opossum | Ja | DD | Unk | 0.03 | 77% |
| Monodelphis ronaldi | Ronald's opossum | Ja | LC | Stable |  | 100% |
| **Diprotodontia (diprotodonts)** | | | | | | |
| Macropus giganteus | Eastern grey kangaroo | Di | LC | Stable | 33.41 | 5% |
| Macropus rufus | Red kangaroo | Di* | LC | Stable | 38.97 | 5% |
| Macropus robustus | Barrow island euro | Di* | LC | Stable | 25.98 | 6% |
| Trichosurus vulpecula | Common brushtail possum | Di | LC | Dec | 2.69 | 8% |
| Wallabia bicolor | Swamp wallaby | Di* | LC | Inc | 15.00 | 8% |
| Petrogale rothschildi | Rothschild's rock wallaby | Di | LC | Unk | 4.55 | 9% |
| Petauroides volans | Greater glider | Di | LC | Dec | 1.26 | 9% |
| Macropus rufogriseus | Bennett's wallaby | Di* | LC | Stable | 16.85 | 10% |
| Pseudocheirus peregrinus | Common ring-tailed possum | Di | LC | Stable | 0.90 | 12% |
| Thylogale thetis | Red-necked pademelon | Di | LC | Stable | 5.40 | 15% |
| Potorous tridactylus | Long-nosed potoroo | Di | LC | Dec | 1.06 | 15% |
| Vombatus ursinus | Coarse-haired wombat | Di | LC | Stable | 26.00 | 18% |
| Macropus parma | Parma wallaby | Di | NT | Unk | 4.16 | 19% |
| Trichosurus cunninghami | Mountain brushtail possum | Di | LC | Stable | NA | 25% |
| Burramys parvus | Broom's pygmy-possum | Di | CR | Dec | 0.04 | 87% |
| **Lagomorpha (rabbits, hares and pikas)** | | | | | | |
| Lepus comus | Yunnan hare | GrWo*,Le,SnLe | LC | Unk | 2.02 | 0% |
| Lepus yarkandensis | Yarkand hare | GrWo*,SnLe | NT | Dec | 1.46 | 0% |
| Ochotona huangensis | Tsing-ling pika | SnLe | LC | Unk | 0.11 | 0% |
| Ochotona cansus | Gansu pika | SnLe | LC | Unk | 0.07 | 0% |
| Ochotona erythrotis | Chinese red pika | SnLe | LC | Unk |  | 0% |
| Ochotona gloveri | Glover's pika | SnLe | LC | Unk |  | 0% |
| Ochotona iliensis | Ili pika | SnLe | EN | Dec |  | 0% |
| Ochotona koslowi | Koslov's pika | SnLe | EN | Dec |  | 0% |
| Ochotona muliensis | Muli pika | SnLe | DD | Unk |  | 0% |
| Ochotona thomasi | Thomas's pika | SnLe | LC | Unk |  | 0% |
| Lepus tibetanus | Desert hare | GrWo*,SnLe | LC | Unk |  | 0% |
| Lepus sinensis | Chinese hare | GrWo*,Le | LC | Unk | 1.61 | 0% |
| Lepus oiostolus | Woolly hare | GrWo*,Le,SnLe | LC | Unk | 2.47 | 0% |
| Ochotona ladacensis | Ladak pika | SnLe | LC | Unk |  | 0% |
| Ochotona curzoniae | Black-lipped pika | SnLe | LC | Dec | 0.16 | 0% |
| Ochotona rufescens | Afghan pika | SnLe | LC | Stable | 0.25 | 0% |
| Ochotona himalayana | Himalayan pika | SnLe | LC | Unk |  | 0% |
| Ochotona pusilla | Little pika | SnLe | LC | Dec | 0.14 | 1% |
| Lepus coreanus | Korean hare | GrWo*,Le | LC | Unk |  | 1% |
| Lepus saxatilis | Savannah hare | Ch,Le | LC | Dec | 2.59 | 1% |
| Lepus habessinicus | Abyssinian hare | Ch,Le | LC | Unk | 2.02 | 1% |
| Lepus alleni | Antelope jackrabbit | GrWo* | LC | Stable | 3.93 | 1% |
| Lepus mandshuricus | Manchurian hare | GrWo*,Le | LC | Unk | 1.83 | 1% |
| Ochotona nubrica | Nubra pika | SnLe | LC | Unk |  | 1% |
| Lepus nigricollis | Black-napped hare | GrWo*,Le,SnLe | LC | Unk | 2.29 | 1% |
| Sylvilagus floridanus | Eastern cottontail | ReWo* | LC | Inc | 1.21 | 1% |
| Lepus granatensis | Granada hare | GrWo* | LC | Stable | 2.32 | 1% |
| Ochotona thibetana | Moupin pika | SnLe | LC | Unk |  | 2% |
| Sylvilagus palustris | Key rabbit | ReWo* | LC | Unk | 1.36 | 2% |
| Ochotona macrotis | Large-eared pika | SnLe | LC | Unk | 0.21 | 2% |
| Lepus capensis | Arabian hare | Ch,EuLy,GrWo*,Le,SnLe | LC | Dec | 2.05 | 2% |
| Ochotona hyperborea | Northern pika | SnLe | LC | Unk | 0.12 | 3% |
| Lepus starcki | Ethiopian highland hare | Ch,EtWo,Le | LC | Unk | 2.76 | 3% |
| Lepus europaeus | Brown hare | GrWo*,Le,Pu,SnLe | LC | Dec | 3.82 | 3% |
| Lepus timidus | Arctic hare | EuLy,GrWo*,Le,SnLe | LC | Unk | 3.11 | 3% |
| Lepus californicus | Black-tailed jackrabbit | GrWo* | LC | Stable | 2.42 | 3% |
| Lepus tolai | Tolai hare | Ch,GrWo*,Le,SnLe | LC | Unk | 1.59 | 4% |
| Lepus fagani | Ethiopian hare | Ch,Le | DD | Unk |  | 4% |
| Ochotona dauurica | Daurian pika | SnLe | LC | Unk | 0.13 | 4% |
| Lepus townsendii | White-tailed jackrabbit | GrWo* | LC | Dec | 3.37 | 4% |
| Lepus microtis | African savanna hare | Ch,Le | LC | Unk | 1.76 | 5% |
| Ochotona roylei | Royle's pika | SnLe | LC | Stable | 0.26 | 5% |
| Oryctolagus cuniculus | European rabbit | Di* | NT | Dec | 1.59 | 5% |
| Ochotona forresti | Forrest's pika | SnLe | LC | Dec |  | 6% |
| Ochotona rutila | Turkestan red pika | SnLe | LC | Stable |  | 6% |
| Lepus othus | Alaskan hare | GrWo* | LC | Unk | 4.84 | 6% |
| Lepus peguensis | Burmese hare | GrWo*,Le | LC | Stable | 2.27 | 7% |
| Ochotona alpina | Alpine pika | SnLe | LC | Unk | 0.15 | 8% |
| Ochotona pallasi | Mongolian pika | SnLe | LC | Dec |  | 9% |
| Lepus americanus | Snowshoe hare | GrWo* | LC | Stable | 1.57 | 9% |
| Lepus corsicanus | Apennine hare | GrWo* | VU | Dec |  | 11% |
| Lepus arcticus | Arctic hare | GrWo* | LC | Unk | 4.41 | 17% |
| Lepus castroviejoi | Broom hare | GrWo* | VU | Dec | 2.82 | 23% |
| **Monotremata (platypus and echidnas)** | | | | | | |
| Tachyglossus aculeatus | Kangaroo island echidna | Di | LC | Stable | 4.50 | 7% |
| **Peramelemorphia (bilbies and bandicoots)** | | | | | | |
| Perameles nasuta | Long-nosed bandicoot | Di | LC | Unk | 0.72 | 15% |
| Isoodon obesulus | Nuyts southern brown bandicoot | Di | LC | Dec | 0.83 | 19% |
| **Perissodactyla (odd-toed ungulates)** | | | | | | |
| Equus grevyi | Grevy's zebra | AfWiDo,Ch,Li*,SpHy | EN | Stable | 408.00 | 4% |
| Tapirus bairdii | Baird's tapir | Ja | EN | Dec | 293.78 | 5% |
| Diceros bicornis | Black rhinoceros | Ch,Le,Li | CR | Inc | 995.94 | 5% |
| Ceratotherium simum | Northern white rhinoceros | Ch,Le,Li | NT | Inc | 2285.94 | 7% |
| Tapirus terrestris | Brazilian tapir | Ja | VU | Dec | 169.50 | 7% |
| Equus zebra | Hartmann's mountain zebra | AfWiDo,Ch,Le,Li*,SpHy | VU | Unk | 282.46 | 11% |
| Equus quagga | Burchell's zebra | AfWiDo,Ch,Le,Li*,SpHy | LC | Stable | 400.00 | 11% |
| Tapirus pinchaque | Andean tapir | Ja | EN | Dec | 156.92 | 13% |
| Rhinoceros unicornis | Greater one-horned rhino | Ti | VU | Inc | 1843.66 | 50% |
| **Pholidota (pangolins)** | | | | | | |
| Manis javanica | Malayan pangolin | ClLe | EN | Dec | 4.86 | 8% |
| **Primates (primates)** | | | | | | |
| Macaca thibetana | Milne-edwards’ macaque | Ti | NT | Dec | 10.59 | 0% |
| Trachypithecus francoisi | Francois's langur | Dh,Le | EN | Dec | 8.14 | 0% |
| Cercopithecus solatus | Sun-tailed guenon | Le | VU | Unk | 5.26 | 0% |
| Cercopithecus dryas | Dryad monkey | Le | CR | Unk | 2.78 | 0% |
| Macaca munzala | Arunachal macaque | Ti | EN | Dec |  | 0% |
| Rungwecebus kipunji | Kipunji | Le | CR | Dec |  | 0% |
| Semnopithecus ajax | Chamba sacred langur | Le | EN | Dec |  | 0% |
| Trachypithecus laotum | Lao langur | Dh,Le,Ti | VU | Dec |  | 0% |
| Semnopithecus dussumieri | Dussumier's malabar langur | Dh,Le,Ti | LC | Stable |  | 0% |
| Macaca radiata | Bonnet macaque | Ti | LC | Dec | 5.00 | 0% |
| Procolobus pennantii | Bouvier's red colobus | Le | CR | Dec |  | 1% |
| Papio hamadryas | Hamadryas baboon | Ch,Le,Li,SpHy | LC | Inc | 14.01 | 1% |
| Semnopithecus entellus | Bengal hanuman langur | Dh,Le,Ti | LC | Dec | 12.68 | 1% |
| Trachypithecus pileatus | Bonneted langur | Dh,Le,Ti | VU | Dec | 11.22 | 1% |
| Macaca mulatta | Rhesus macaque | SnLe,Ti | LC | Unk | 6.46 | 1% |
| Cercopithecus erythrogaster | Red-bellied guenon | Le | VU | Dec | 3.45 | 1% |
| Nycticebus pygmaeus | Lesser slow loris | ClLe | VU | Dec | 0.34 | 2% |
| Colobus polykomos | King colobus | Le | VU | Unk | 8.80 | 2% |
| Cercopithecus diana | Diana guenon | Le | VU | Dec | 4.36 | 2% |
| Papio papio | Guinea baboon | Le,Li,SpHy | NT | Unk | 18.03 | 2% |
| Colobus satanas | Black colobus | Le | VU | Dec | 9.06 | 2% |
| Cercopithecus mona | Mona guenon | Le | LC | Unk | 3.98 | 3% |
| Trachypithecus auratus | Ebony leaf monkey | Dh,Le | VU | Dec | 9.72 | 3% |
| Cercopithecus petaurista | Lesser spot-nosed guenon | Le | LC | Unk | 3.23 | 3% |
| Procolobus badius | Bay colobus | Le | EN | Dec |  | 3% |
| Trachypithecus delacouri | Delacour's langur | Dh,Le,Ti | CR | Dec |  | 3% |
| Macaca assamensis | Assamese macaque | Ti | NT | Dec | 8.55 | 3% |
| Semnopithecus hector | Gray langur | Dh,Le,Ti | NT | Dec |  | 3% |
| Cercocebus atys | Red-capped monkey | Le | VU | Dec | 6.94 | 3% |
| Trachypithecus poliocephalus | Cat ba langur | Dh,Le | CR | Dec |  | 3% |
| Colobus angolensis | Angola colobus | Le | LC | Unk | 8.99 | 3% |
| Cercocebus torquatus | Collared mangabey | Le | VU | Dec | 7.29 | 4% |
| Trachypithecus shortridgei | Shortridge’s capped langur | Dh,Le,Ti | EN | Dec |  | 4% |
| Cercopithecus nictitans | Greater spot-nosed guenon | Le | LC | Dec | 5.26 | 4% |
| Cercopithecus campbelli | Campbell's guenon | Le | LC | Unk | 3.63 | 4% |
| Papio anubis | Anubis baboon | Ch,Le,Li,SpHy | LC | Inc | 17.73 | 4% |
| Cercopithecus ascanius | Black-cheeked white-nosed monkey | Le | LC | Unk | 3.54 | 4% |
| Cercopithecus cephus | Moustached guenon | Le | LC | Unk | 3.45 | 4% |
| Presbytis siamensis | Pale-thighed langur | Dh,Le,Ti | NT | Dec | 5.90 | 4% |
| Macaca silenus | Lion-tailed macaque | Ti | EN | Dec | 6.00 | 4% |
| Cercopithecus neglectus | De brazza's monkey | Le | LC | Unk | 5.33 | 4% |
| Semnopithecus schistaceus | Central himalayan langur | Dh,Le,Ti | LC | Dec |  | 4% |
| Papio ursinus | Chacma baboon | Ch,Le,Li,SpHy | LC | Stable | 17.73 | 4% |
| Lophocebus aterrimus | Black crested mangabey | Le | NT | Dec | 6.51 | 5% |
| Papio cynocephalus | Yellow baboon | Ch,Le,Li,SpHy | LC | Stable | 15.82 | 5% |
| Trachypithecus phayrei | Phayre's langur | Dh,Le,Ti | EN | Dec | 7.68 | 5% |
| Semnopithecus priam | Coromandel sacred langur | Dh,Le,Ti | NT | Dec |  | 5% |
| Gorilla gorilla | Lowland gorilla | Le | CR | Dec | 112.59 | 5% |
| Macaca arctoides | Bear macaque | Ti | VU | Dec | 9.36 | 5% |
| Colobus vellerosus | Geoffroy's black-and-white colobus | Le | VU | Unk | 7.70 | 5% |
| Nycticebus bengalensis | Bengal loris | ClLe | VU | Dec | 1.14 | 5% |
| Pan troglodytes | Chimpanzee | Le | EN | Dec | 45.00 | 5% |
| Lophocebus albigena | Gray-cheeked mangabey | Le | LC | Dec | 7.42 | 5% |
| Trachypithecus johnii | Black leaf monkey | Dh,Le,Ti | VU | Dec | 10.60 | 5% |
| Cercopithecus mitis | Blue monkey | Le | LC | Dec | 5.04 | 5% |
| Chlorocebus aethiops | Green monkey | Ch,Le | LC | Stable | 3.70 | 6% |
| Trachypithecus germaini | Germain’s langur | Dh,Le,Ti | EN | Dec |  | 6% |
| Trachypithecus hatinhensis | Hatinh langur | Dh,Le,Ti | EN | Dec |  | 6% |
| Colobus guereza | Eastern black-and-white colobus | Le | LC | Unk | 9.93 | 6% |
| Cercopithecus pogonias | Crowned guenon | Le | LC | Unk | 3.58 | 6% |
| Nasalis larvatus | Long-nosed monkey | SuClLe | EN | Dec | 12.27 | 6% |
| Cercocebus agilis | Agile mangabey | Le | LC | Stable | 7.11 | 6% |
| Macaca fascicularis | Crab-eating macaque | Ti | LC | Dec | 4.57 | 6% |
| Presbytis comata | Grizzled leaf monkey | Dh,Le | EN | Dec | 6.55 | 6% |
| Macaca leonina | Northern pig-tailed macaque | Ti | VU | Dec |  | 6% |
| Trachypithecus cristatus | Silvered langur | Dh,Le,Ti | NT | Dec | 7.18 | 7% |
| Procolobus rufomitratus | Eastern red colobus | Le | LC | Dec |  | 7% |
| Macaca nemestrina | Pig-tailed macaque | Ti | VU | Dec | 7.82 | 7% |
| Cercocebus chrysogaster | Golden-bellied mangabey | Le | DD | Dec |  | 7% |
| Nycticebus coucang | Greater slow loris | ClLe | VU | Dec | 0.93 | 8% |
| Semnopithecus hypoleucos | Black-footed gray langur | Dh,Le,Ti | VU | Dec | 10.03 | 8% |
| Presbytis hosei | Gray leaf monkey | SuClLe | VU | Dec | 6.28 | 8% |
| Presbytis melalophos | Mitred leaf monkey | Dh,Ti | EN | Dec | 6.44 | 8% |
| Cercopithecus erythrotis | Red-eared guenon | Le | VU | Dec | 3.26 | 10% |
| Cercopithecus preussi | Preuss's guenon | Le | EN | Dec | 5.13 | 11% |
| Trachypithecus obscurus | Dusky langur | Dh,Le,Ti | NT | Dec | 7.25 | 12% |
| Presbytis thomasi | North sumatran leaf monkey | Dh,Ti | VU | Dec | 6.69 | 13% |
| Cercopithecus hamlyni | Hamlyn’s monkey | Le | VU | Dec |  | 13% |
| Cercopithecus lhoesti | L'hoest's guenon | Le | VU | Dec | 5.31 | 14% |
| Presbytis femoralis | Banded langur | Dh,Le,Ti | NT | Dec | 7.03 | 15% |
| Cercocebus galeritus | Tana river crested mangabey | Le | EN | Dec | 7.08 | 16% |
| Trachypithecus vetulus | Purple-faced langur | Le | EN | Dec | 7.21 | 16% |
| Trachypithecus geei | Gee's golden langur | Dh,Le,Ti | EN | Dec | 8.36 | 25% |
| Procolobus gordonorum | Udzungwa red colobus | Le | EN | Dec |  | 30% |
| Procolobus preussi | Preuss's red colobus | Le | CR | Dec |  | 35% |
| Trachypithecus barbei | Barbe's langur | Dh,Le,Ti | DD | Dec |  | 41% |
| Cercocebus sanjei | Sanje crested mangabey | Le | EN | Dec |  | 53% |
| **Proboscidea (elephants)** | | | | | | |
| Elephas maximus | Asian elephant | Ti | EN | Dec | 3269.79 | 11% |
| Loxodonta africana | African elephant | Ch,Le,Li,SpHy | VU | Inc | 3824.54 | 21% |
| **Rodentia (rodents)** | | | | | | |
| Notomys aquilo | Northern hopping mouse | Di | EN | Dec | 0.04 | 0% |
| Aeretes melanopterus | Groove-toothed flying squirrel | SnLe | NT | Dec |  | 0% |
| Biswamoyopterus biswasi | Namdapha flying squirrel | SnLe | CR | Dec |  | 0% |
| Eupetaurus cinereus | Woolly flying squirrel | SnLe | EN | Unk |  | 0% |
| Sciurotamias davidianus | Pére david's rock squirrel | Le,SnLe | LC | Unk |  | 0% |
| Sciurotamias forresti | Forrest's rock squirrel | Le | LC | Unk |  | 0% |
| Spermophilus taurensis |  | Le | LC | Unk |  | 0% |
| Spermophilus xanthoprymnus | Asia minor ground squirrel | Le | NT | Dec |  | 0% |
| Trogopterus xanthipes | Complex-toothed flying squirrel | SnLe | NT | Dec |  | 0% |
| Petaurista xanthotis | Chinese giant flying squirrel | SnLe | LC | Unk |  | 0% |
| Spermophilus ralli | Tien shan ground squirrel | SnLe | LC | Unk |  | 0% |
| Petaurista alborufus | Red and white giant flying squirrel | SnLe | LC | Unk | 1.50 | 0% |
| Spermophilus fulvus | Yellow ground squirrel | Le,SnLe | LC | Unk | 0.78 | 0% |
| Dremomys pernyi | Perny's long-nosed squirrel | SnLe | LC | Stable | 0.20 | 0% |
| Spermophilus pygmaeus | Little ground squirrel | Le | LC | Dec | 0.17 | 0% |
| Tamiops swinhoei | Swinhoe's striped squirrel | SnLe | LC | Stable |  | 1% |
| Sigmodon hispidus | Hispid cotton rat | ReWo | LC | Inc | 0.11 | 1% |
| Funambulus pennantii | Five-striped palm squirrel | SnLe | LC | Unk | 0.10 | 1% |
| Spermophilus dauricus | Daurian ground squirrel | Le | LC | Unk |  | 1% |
| Marmota himalayana | Himalayan marmot | Le,SnLe | LC | Unk |  | 1% |
| Callosciurus pygerythrus | Hoary-bellied squirrel | SnLe | LC | Unk |  | 1% |
| Spermophilopsis leptodactylus | Long-clawed ground squirrel | SnLe | LC | Unk |  | 1% |
| Spermophilus brevicauda | Brandt's ground squirrel | SnLe | LC | Unk |  | 1% |
| Spermophilus major | Russet ground squirrel | SnLe | LC | Unk |  | 1% |
| Spermophilus erythrogenys | Red-cheeked ground squirrel | SnLe | LC | Stable |  | 1% |
| Otomys typus | Typical vlei rat | EtWo* | LC | Dec |  | 1% |
| Spermophilus alashanicus | Alashan ground squirrel | Le,SnLe | LC | Dec |  | 2% |
| Pseudomys australis | Plains mouse | Di | VU | Dec | 0.05 | 2% |
| Myocastor coypus | Coypu | Ja | LC | Dec | 6.36 | 2% |
| Tamias sibiricus | Siberian chipmunk | Le,SnLe | LC | Stable | 0.09 | 2% |
| Pteromys volans | Russian flying squirrel | SnLe | LC | Dec | 0.14 | 2% |
| Callosciurus erythraeus | Pallas's squirrel | SnLe | LC | Stable | 0.28 | 3% |
| Sciurus vulgaris | Eurasian red squirrel | SnLe | LC | Dec | 0.33 | 3% |
| Dremomys lokriah | Orange-bellied himalayan squirrel | SnLe | LC | Dec | 0.23 | 3% |
| Leggadina forresti | Central short-tailed mouse | Di | LC | Unk | 0.02 | 3% |
| Petaurista philippensis | Indian giant flying squirrel | SnLe | LC | Dec | 1.68 | 3% |
| Mus musculus | House mouse | Di,ReWo | LC | Stable | 0.02 | 3% |
| Hylopetes alboniger | Particolored flying squirrel | SnLe | LC | Dec | 0.26 | 3% |
| Hystrix brachyura | Himalayan crestless porcupine | SuClLe | LC | Dec | 8.00 | 4% |
| Rattus villosissimus | Long-haired rat | Di | LC | Unk | 0.22 | 4% |
| Eoglaucomys fimbriatus | Small kashmir flying squirrel | SnLe | LC | Unk | 0.51 | 4% |
| Marmota baibacina | Altai marmot | SnLe | LC | Unk |  | 4% |
| Spermophilus pallidicauda | Pallid ground squirrel | SnLe | LC | Unk |  | 4% |
| Atherurus macrourus | Asiatic brush-tailed porcupine | ClLe | LC | Dec | 2.00 | 4% |
| Pseudomys desertor | Brown desert mouse | Di* | LC | Dec | 0.04 | 5% |
| Petaurista elegans | Grey-headed flying squirrel | SnLe | LC | Stable | 1.04 | 5% |
| Petaurista petaurista | Common giant flying squirrel | SnLe | LC | Dec | 1.53 | 5% |
| Marmota caudata | Long-tailed marmot | Le,SnLe | LC | Unk | 4.35 | 5% |
| Dremomys rufigenis | Asian red-cheeked squirrel | SnLe | LC | Stable | 0.20 | 5% |
| Belomys pearsonii | Hairy-footed flying squirrel | SnLe | DD | Unk |  | 6% |
| Spermophilus undulatus | Long-tailed ground squirrel | SnLe | LC | Stable | 0.74 | 6% |
| Pseudomys hermannsburgensis | Sandy inland mouse | Di | LC | Stable | 0.01 | 6% |
| Hydrochoerus hydrochaeris | Capybara | Ja*,Pu | LC | Unk | 48.15 | 6% |
| Ratufa bicolor | Black giant squirrel | SnLe | NT | Dec | 1.61 | 6% |
| Ondatra zibethicus | Muskrat | ReWo | LC | Stable | 0.99 | 6% |
| Castor canadensis | American beaver | GrWo* | LC | Stable | 18.12 | 7% |
| Cuniculus paca | Spotted paca | Ja* | LC | Stable | 8.17 | 7% |
| Petaurista magnificus | Hodgson's giant flying squirrel | SnLe | LC | Dec | 1.48 | 7% |
| Pedetes capensis | Springhaas | Ch,Le | LC | Unk | 2.55 | 7% |
| Marmota sibirica | Mongolian marmot | SnLe | EN | Dec |  | 7% |
| Alticola roylei | Royle's mountain vole | SnLe | NT | Dec | 0.04 | 7% |
| Tamiops macclellandii | Himalayan striped squirrel | SnLe | LC | Stable |  | 7% |
| Spermophilus relictus | Tien shan ground squirrel | SnLe | LC | Unk | 0.60 | 8% |
| Menetes berdmorei | Indochinese ground squirrel | ClLe | LC | Stable |  | 8% |
| Erethizon dorsatum | North american porcupine | Pu* | LC | Stable | 7.42 | 8% |
| Arvicanthis blicki | Blick's grass rat | EtWo* | NT | Unk | 0.13 | 9% |
| Petaurista nobilis | Bhutan giant flying squirrel | SnLe | NT | Dec |  | 14% |
| Rattus fuscipes | Bush rat | Di | LC | Stable | 0.13 | 18% |
| Spermophilus musicus | Caucasian mountain ground squirrel | Le | NT | Unk |  | 26% |
| Lophuromys melanonyx | Black-clawed brush-furred rat | EtWo* | VU | Unk | 0.01 | 26% |
| Marmota menzbieri | Menzbier's marmot | SnLe | VU | Dec |  | 35% |
| Mastacomys fuscus | Broad-toothed mouse | Di | NT | Dec | 0.13 | 36% |
| Tachyoryctes macrocephalus | Giant mole rat | EtWo* | EN | Dec | 0.62 | 46% |
| **Tubulidentata (aardvark)** | | | | | | |
| Orycteropus afer | Aardvark | Le | LC | Unk | 56.18 | 4% |

**
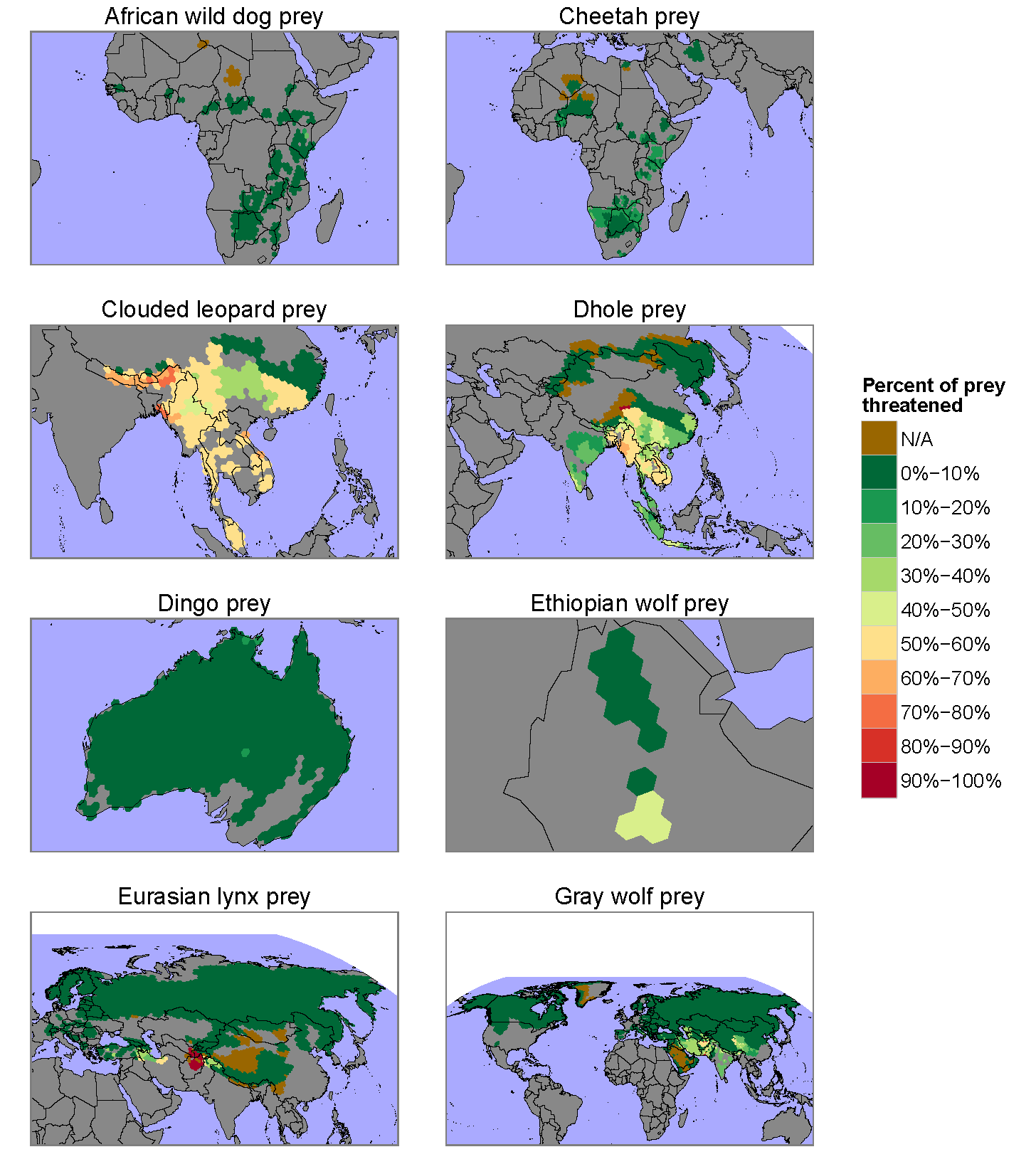
**

**
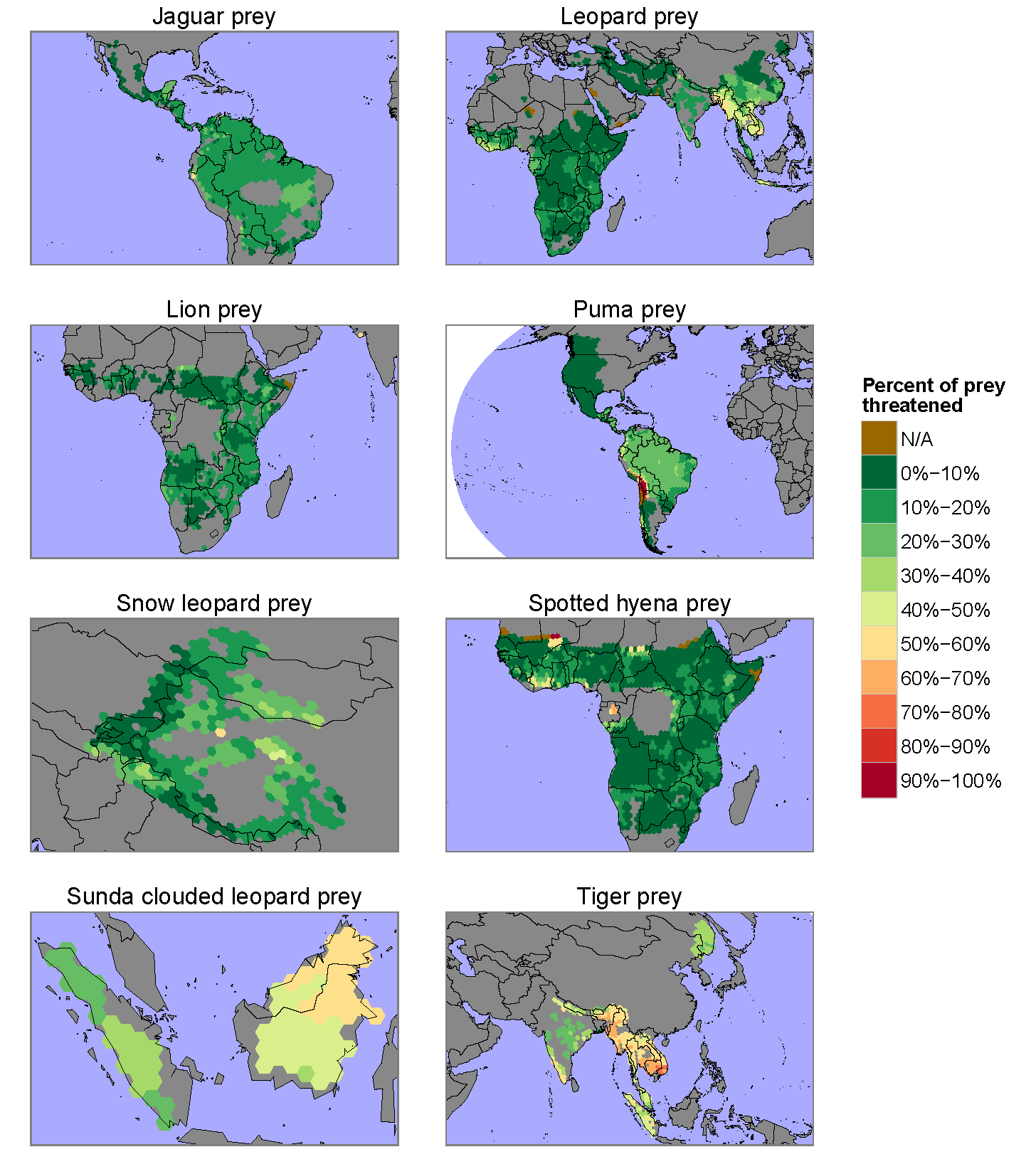
**

**Figure S1.** Prey endangerment maps (complete set).

**
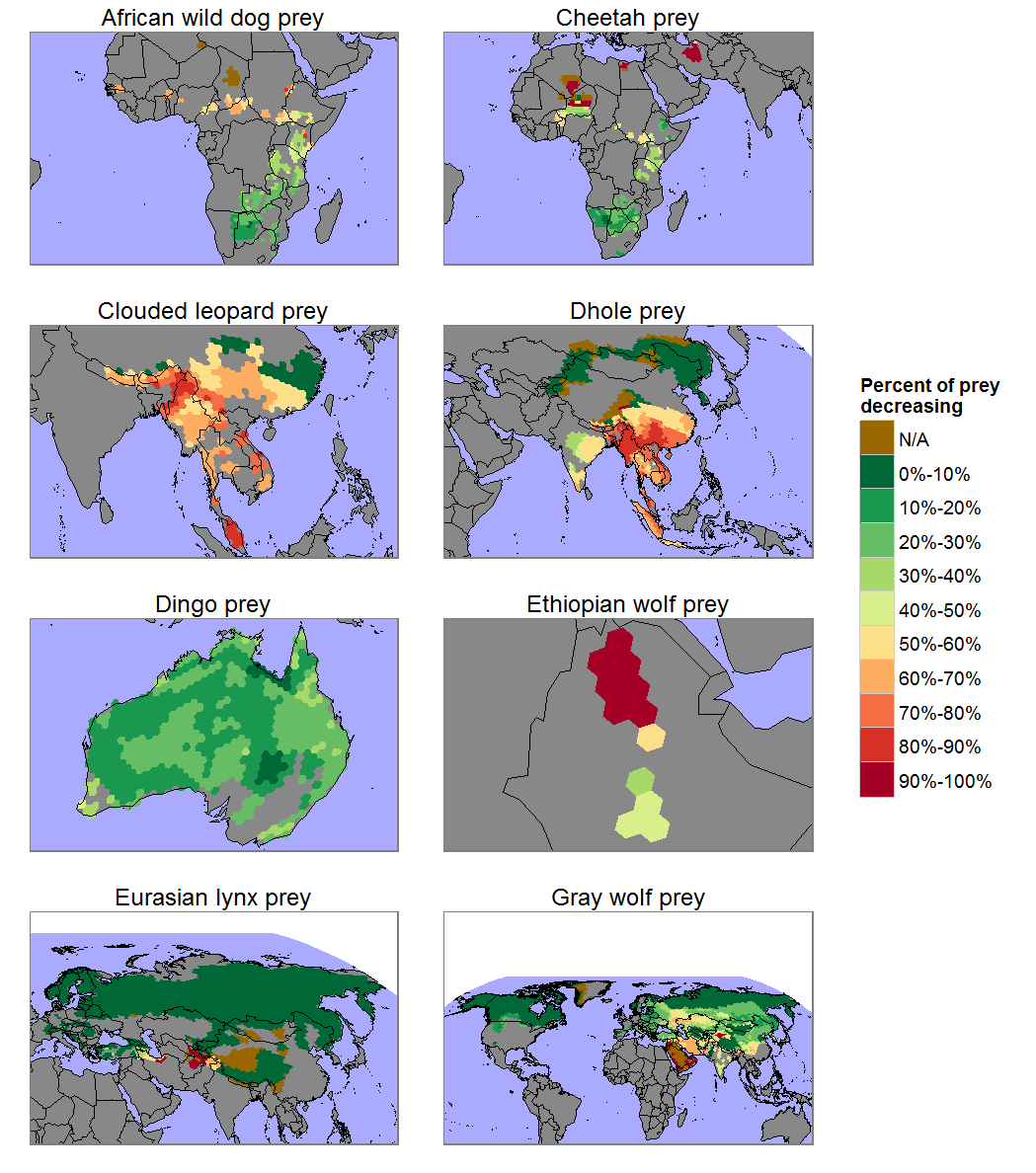

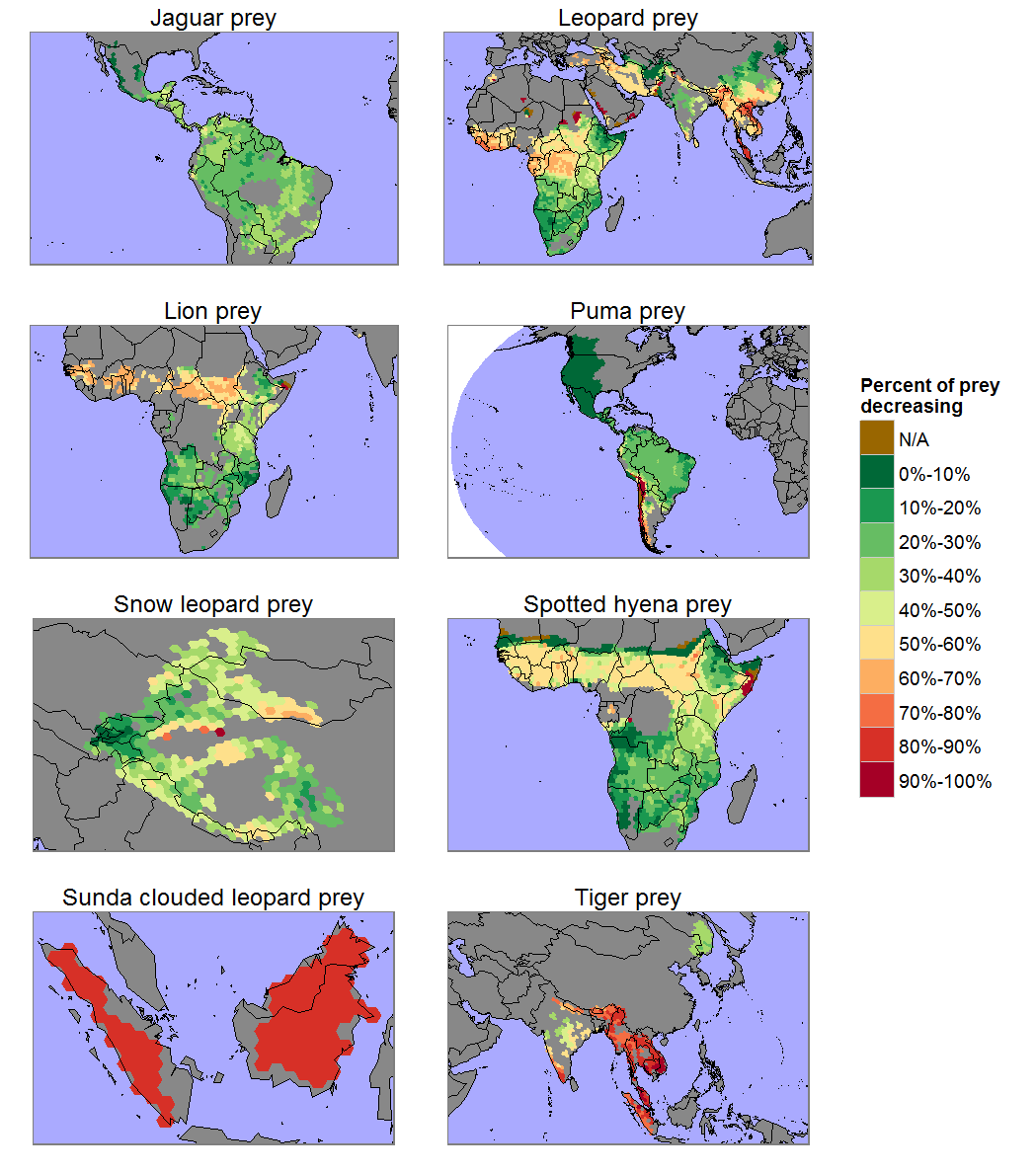
**

**Figure S2.** Prey decreasing trend maps (complete set).

**
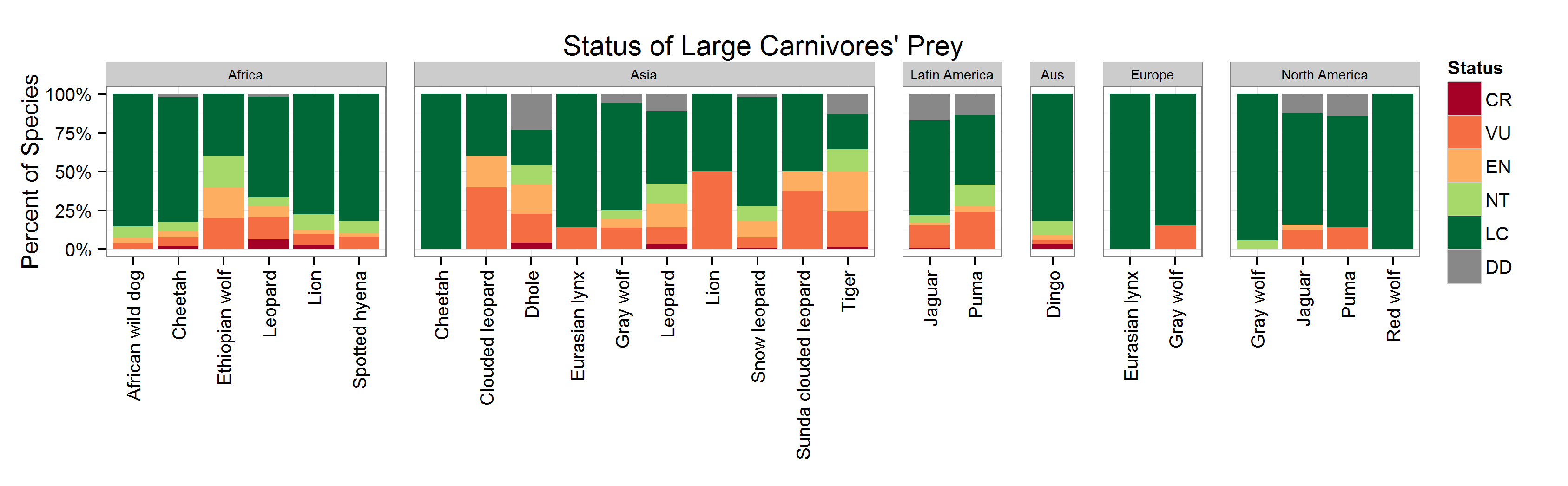
**

**Figure S3.** Prey status by continent. “Status” is the IUCN Red List endangerment status: DD (data deficient), LC (least concern), NT (near threatened), VU (vulnerable), EN (endangered), CR (critically endangered).

**
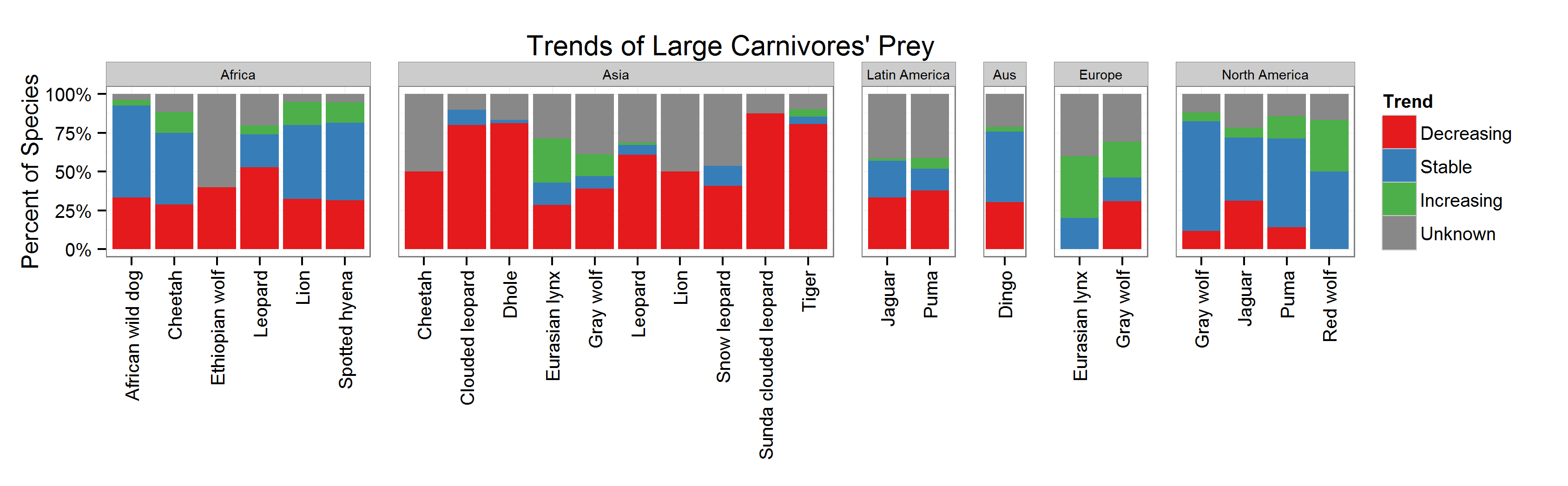
**

**Figure S4.** Prey population trends by continent.

**
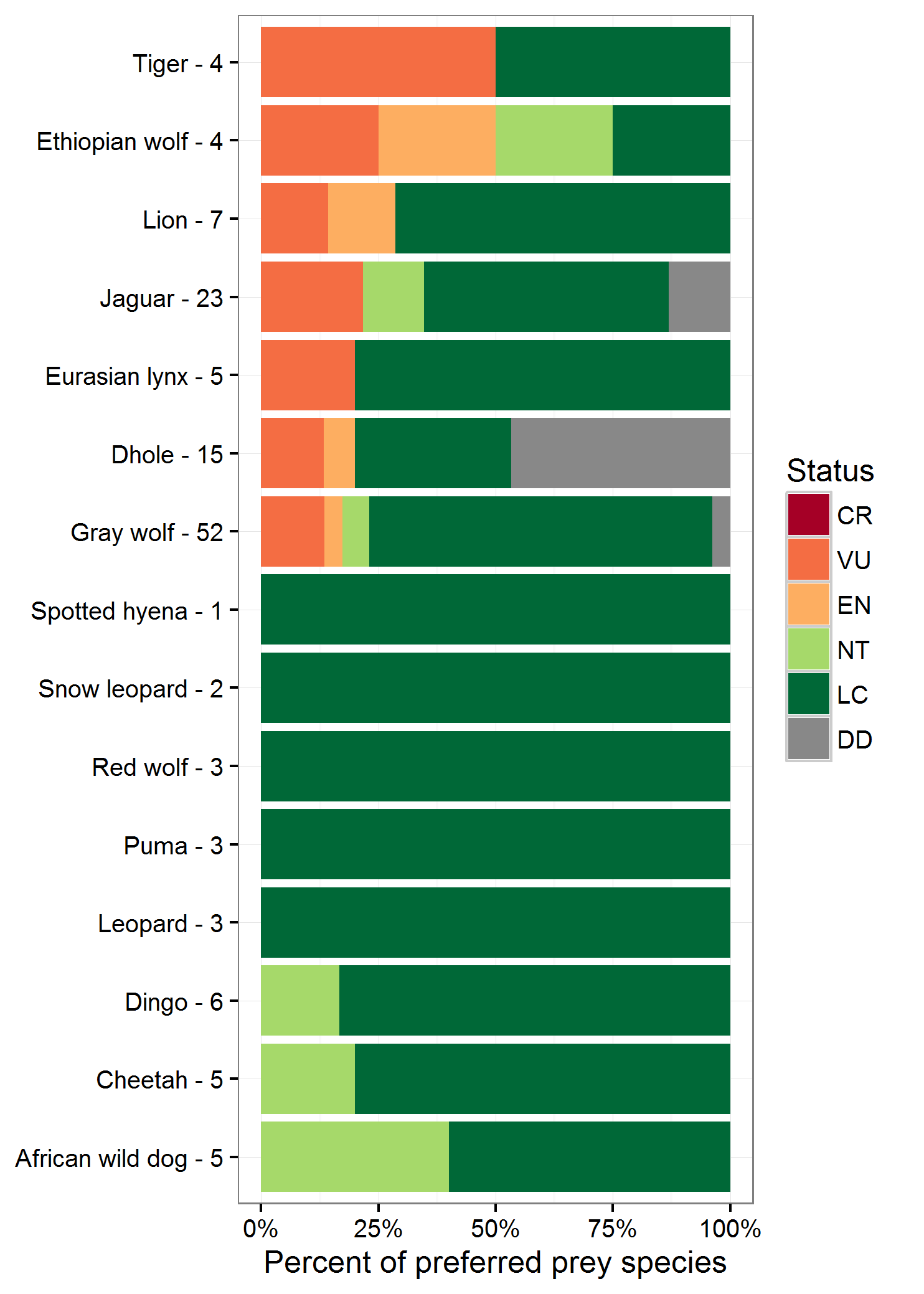
**

**Figure S5.** Status of large carnivores’ preferred prey. Carnivores are ordered by decreasing percentage threatened (VU/EN/CR) prey from the top down. The numbers of preferred prey species are shown after the large carnivore names. “Status” is the IUCN Red List endangerment status: DD (data deficient), LC (least concern), NT (near threatened), VU (vulnerable), EN (endangered), CR (critically endangered).

**
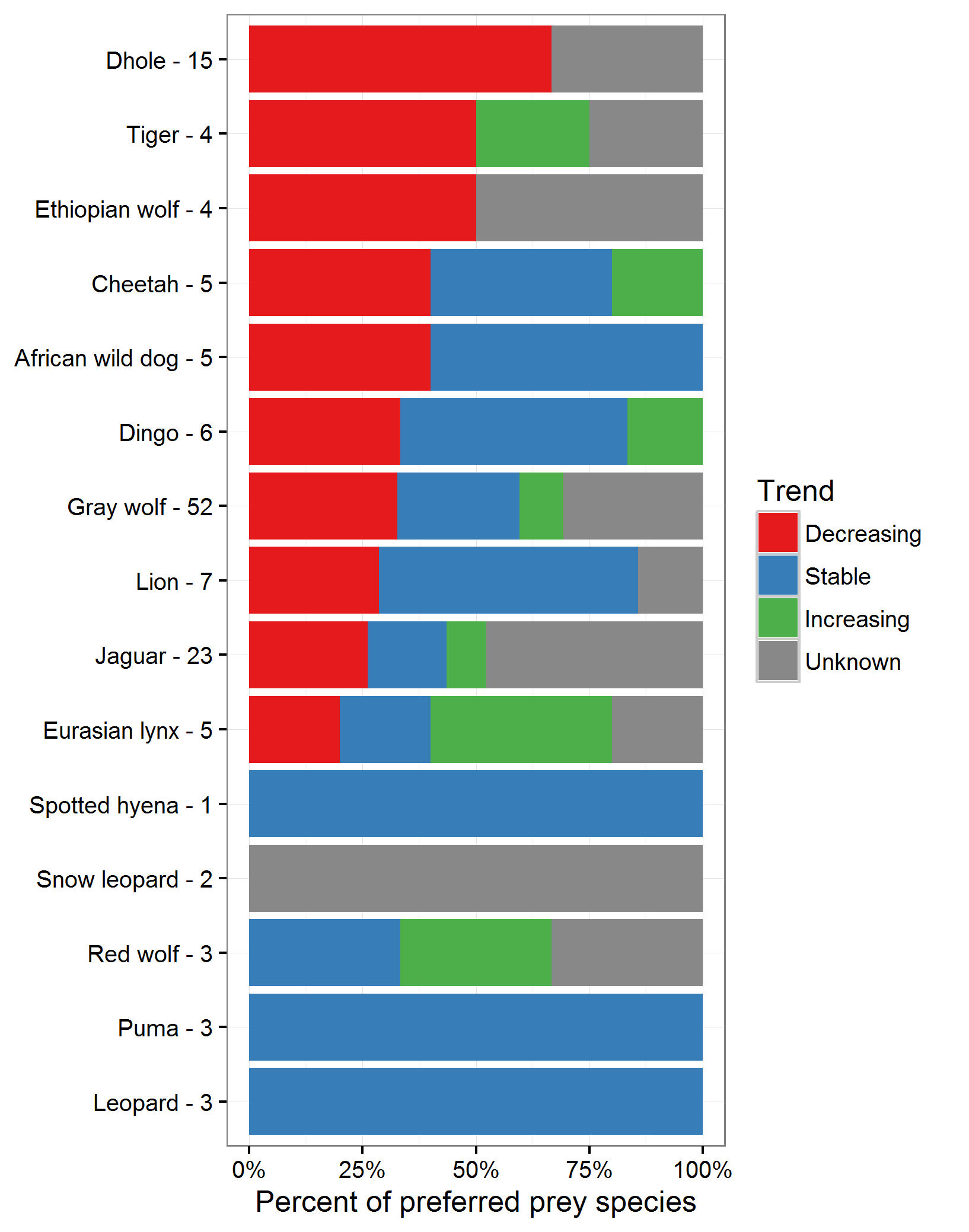
**

**Figure S6.** Population trends of large carnivores’ preferred prey. Carnivores are sorted by percentage of preferred prey with decreasing population trends. The numbers of preferred prey species are shown after the large carnivore names.

**
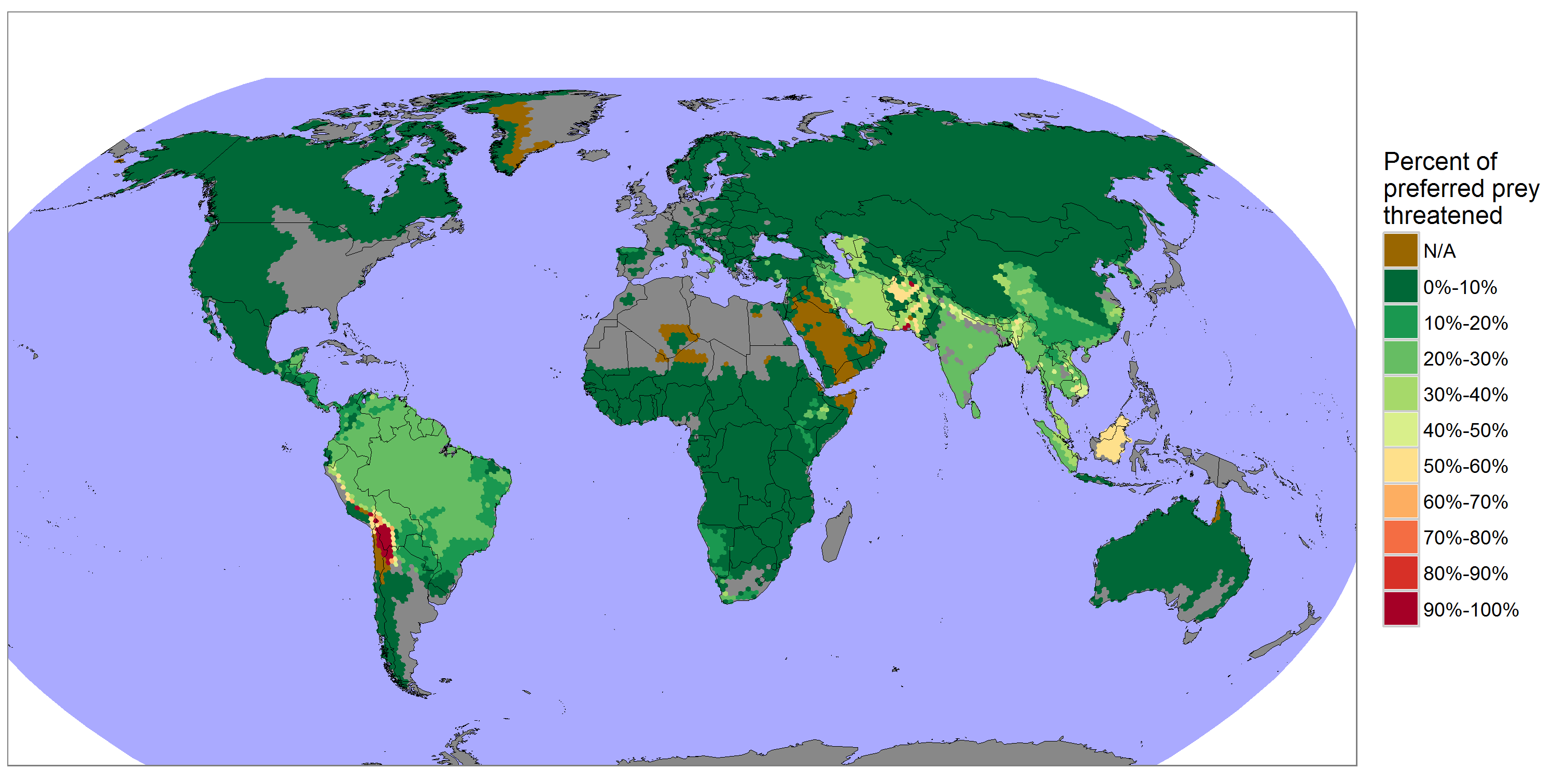
**

**
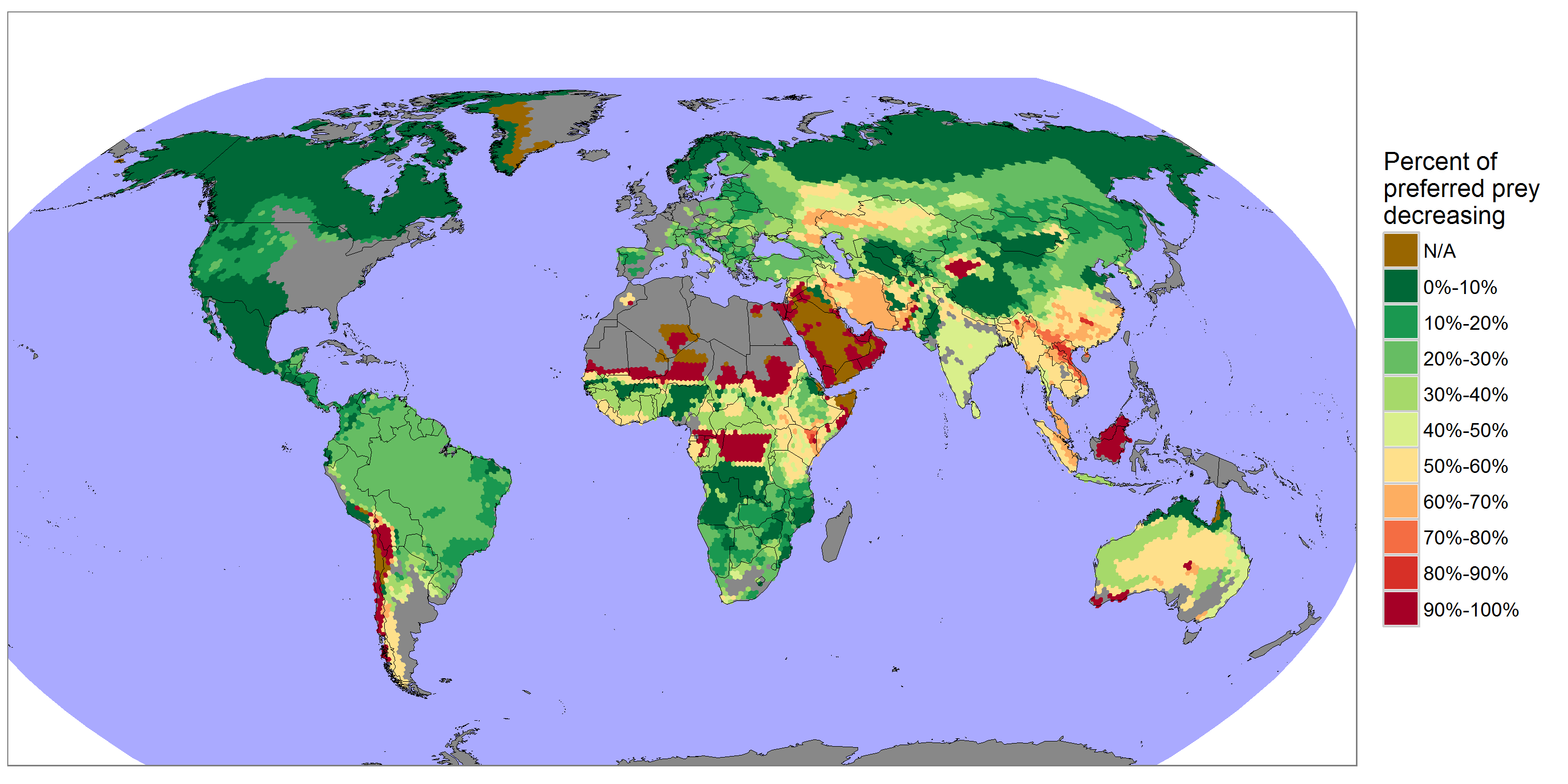
**

**Figure S7.** Percentages of all 114 preferred prey species that are threatened (top) or have decreasing population trends (bottom).

**
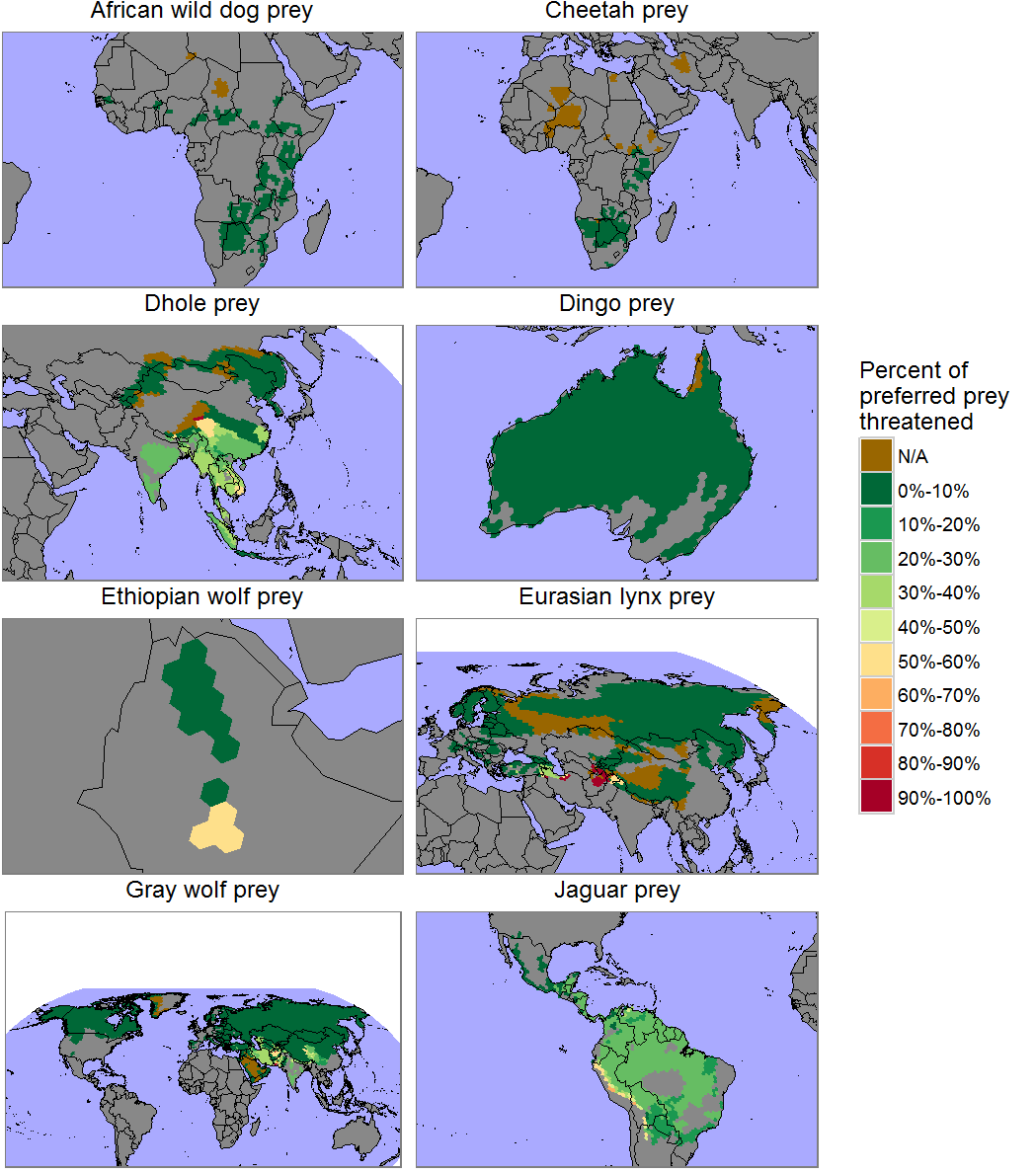

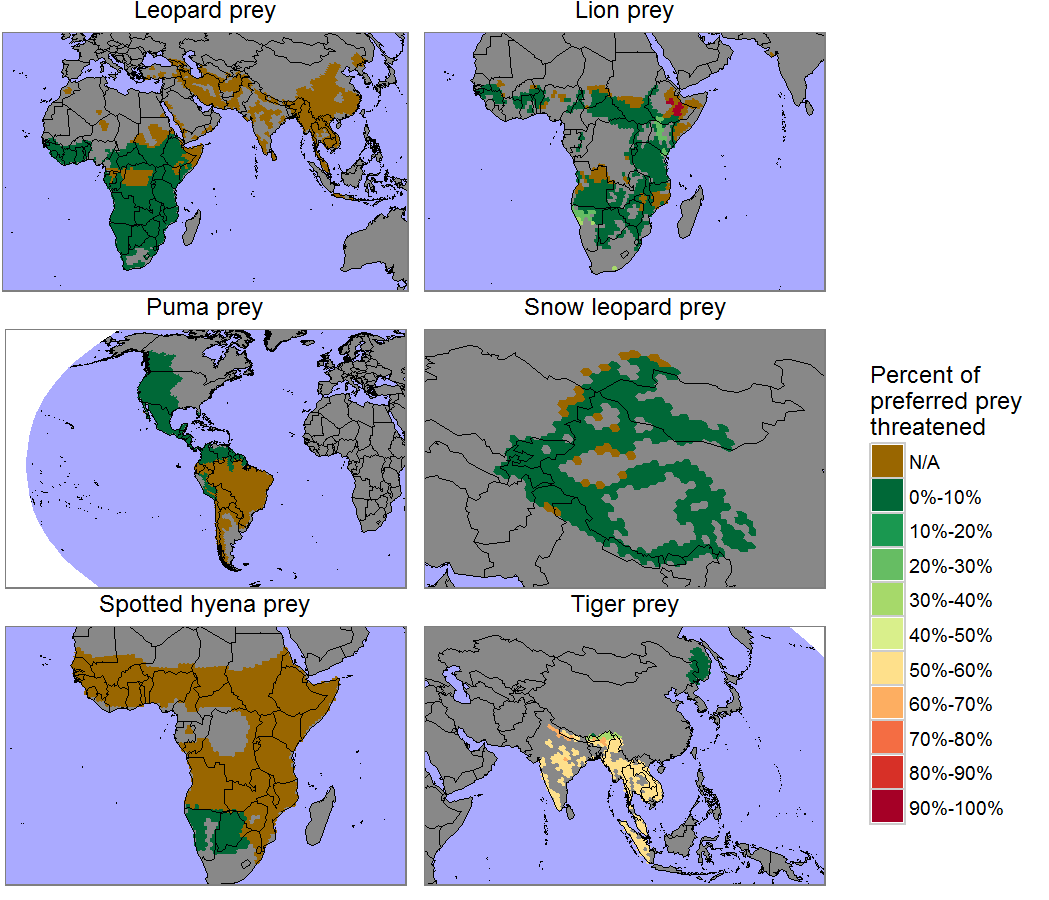
**

**Figure S8.** Preferred prey endangerment maps (complete set).

**
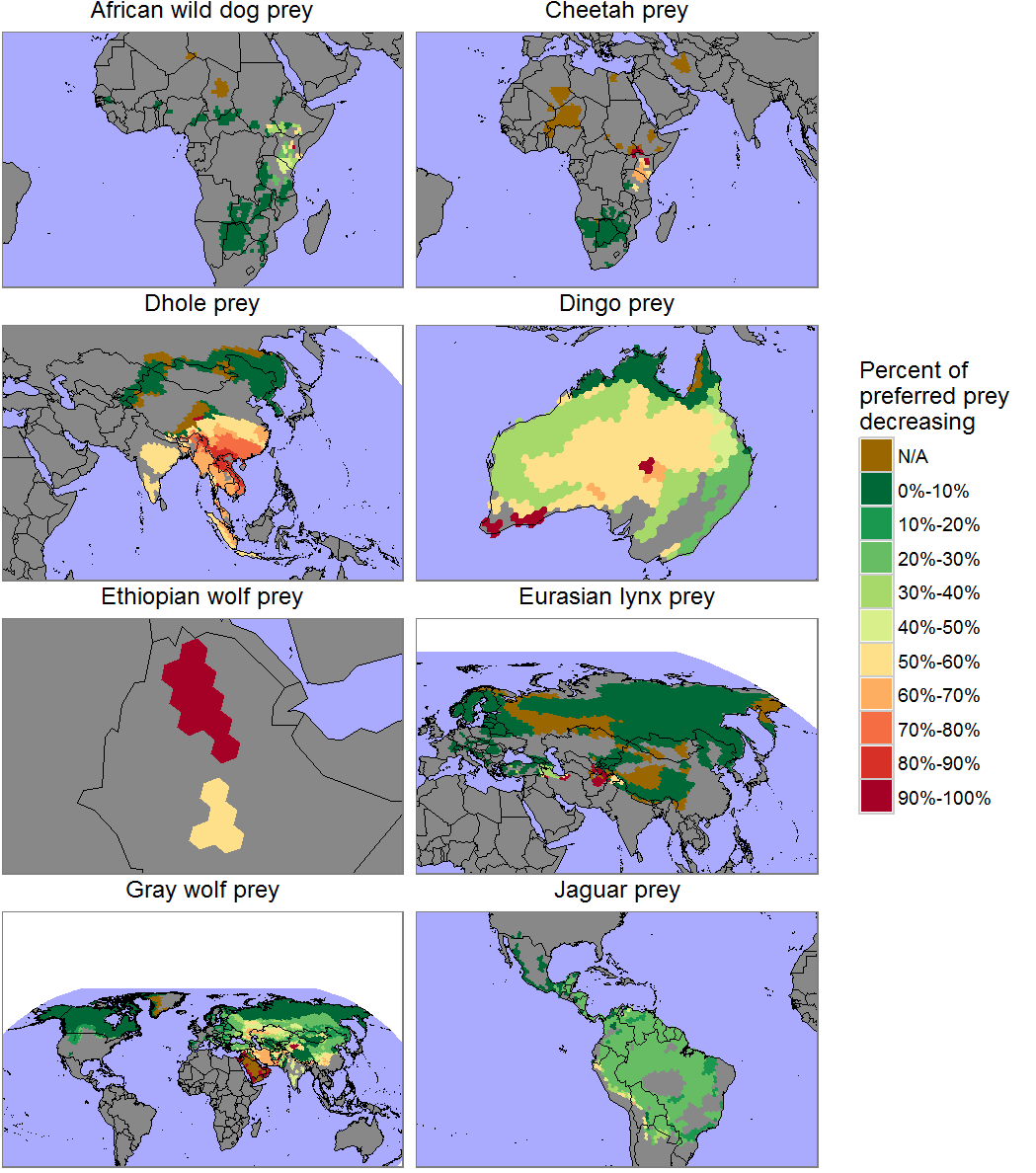
**

**
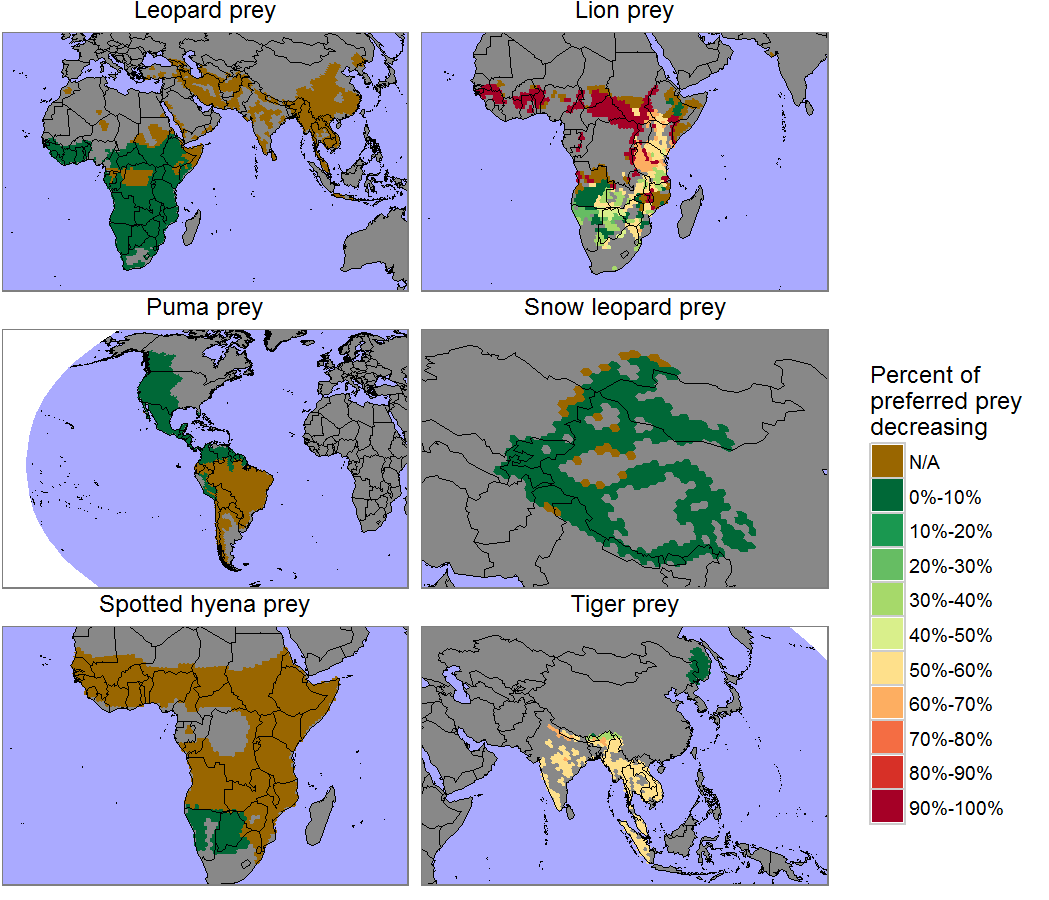
**

**Figure S9.** Preferred prey decreasing trend maps (complete set).


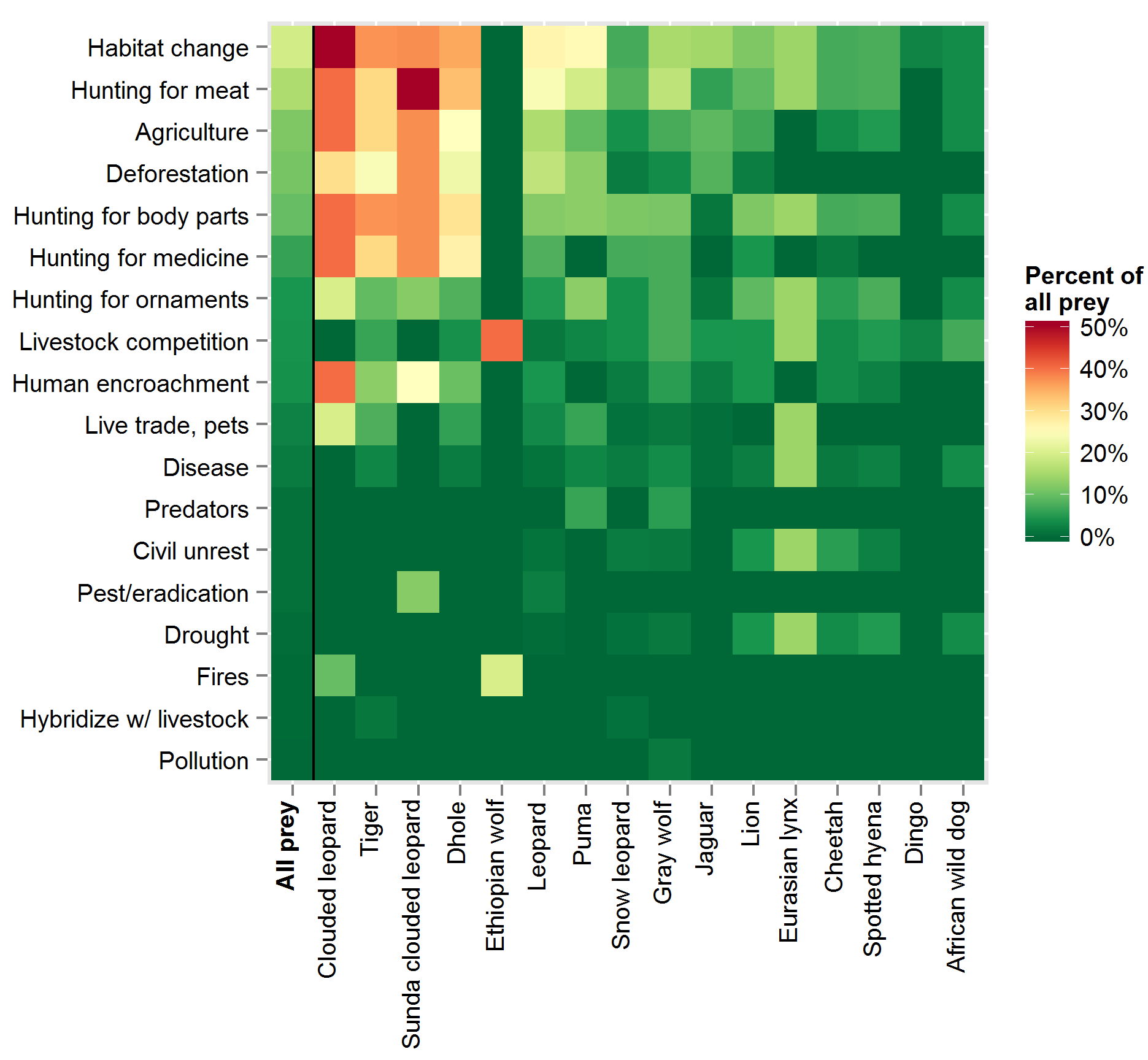


**Figure S10.** Major threats faced by large carnivores’ threatened prey. The percentages are out of all prey, including non-threatened prey. The red wolf is omitted as none of its prey are threatened.


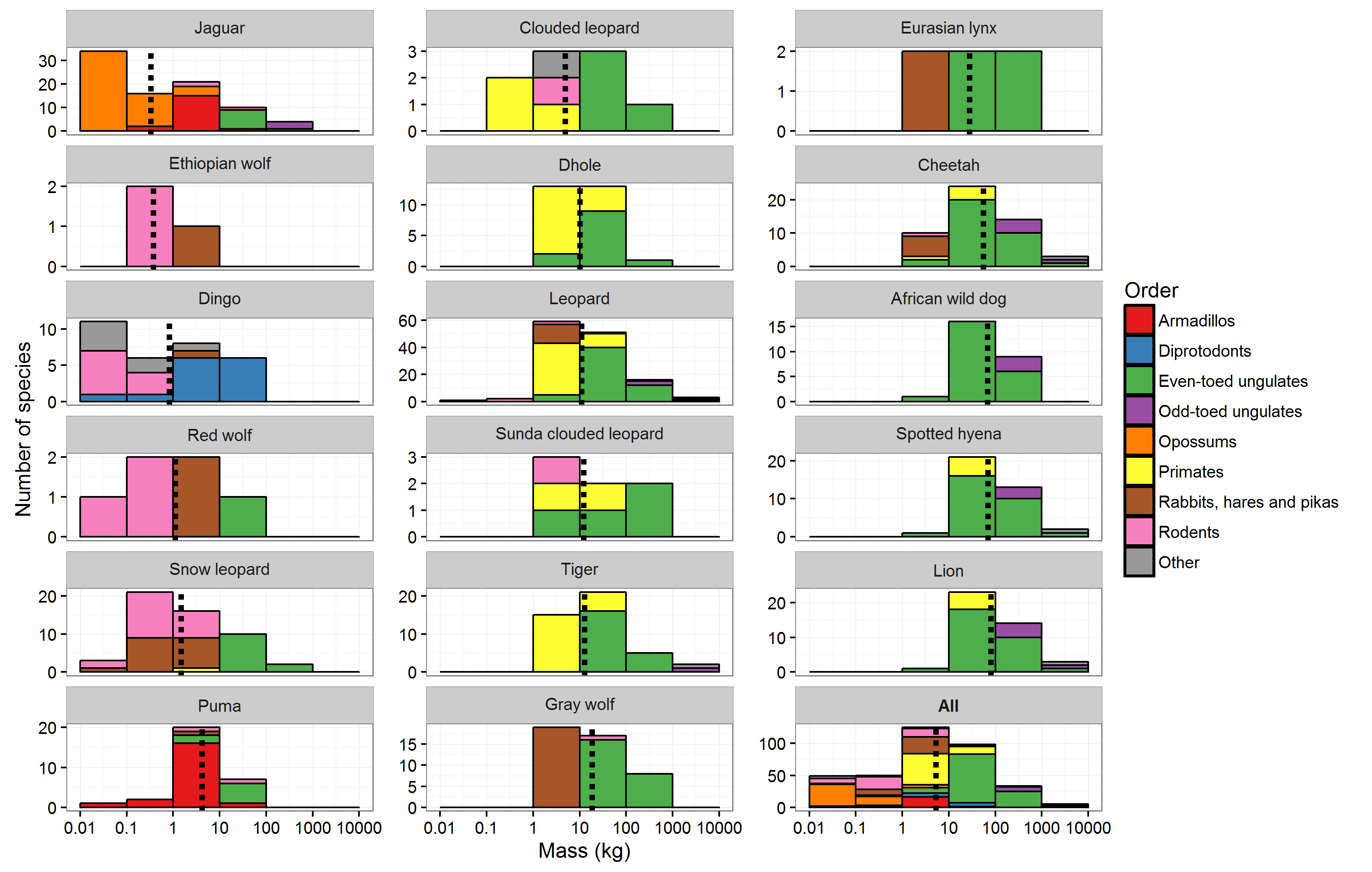


**Figure S11.** Mass and order information for each large carnivore’s prey species. Results for all prey species together are shown in the bottom right panel. Masses were obtained for 360 (73%) of the 494 total prey species from Jones et al. (2009). Only orders with more than five prey species appearing in large carnivore prey lists are labeled. Carnivores are sorted top to bottom, left to right by median prey mass (indicated by vertical lines).

Jones KE, Bielby J, Cardillo M, Fritz SA, O’Dell J, Orme CDL, et al. PanTHERIA: a species-level database of life history, ecology, and geography of extant and recently extinct mammals. Ecology. 2009 Aug 17;90(9):2648–2648.

**Table S3.** Analysis of the six most cited conservation-related articles for each large carnivore. Articles are classified according to whether the term prey was mentioned (“Prey”) and whether prey endangerment, status or depletion was mentioned (“Status”). Only four articles were available for the Sunda clouded leopard.

| **Species** | **Article title** | **Prey** | **Status** |
| --- | --- | --- | --- |
| Cheetah | Laurenson, M. Karen. "High juvenile mortality in cheetahs (Acinonyx jubatus) and its consequences for maternal care." *Journal of Zoology* 234.3 (1994): 387-408. | Y | N |
|  | Merola, Michele. "A reassessment of homozygosity and the case for inbreeding depression in the cheetah, Acinonyx jubatus: implications for conservation." *Conservation biology* 8.4 (1994): 961-971. | Y | Y |
|  | Hayward, M. W., et al. "Prey preferences of the cheetah (Acinonyx jubatus)(Felidae: Carnivora): morphological limitations or the need to capture rapidly consumable prey before kleptoparasites arrive?." *Journal of Zoology* 270.4 (2006): 615-627. | Y | Y |
|  | Kelly, Marcella J., et al. "Demography of the Serengeti cheetah (Acinonyx jubatus) population: the first 25 years." *Journal of Zoology* 244.4 (1998): 473-488. | Y | Y |
|  | Crooks, Kevin R., M. A. Sanjayan, and Daniel F. Doak. "New insights on cheetah conservation through demographic modeling." *Conservation Biology* 12.4 (1998): 889-895. | Y | Y |
|  | Durant, Sarah M. "Predator avoidance, breeding experience and reproductive success in endangered cheetahs, Acinonyx jubatus." *Animal behaviour* 60.1 (2000): 121-130. | Y | N |
| Gray wolf | Mladenoff, David J., et al. "A regional landscape analysis and prediction of favorable gray wolf habitat in the northern Great Lakes region." *Conservation Biology* 9.2 (1995): 279-294. | Y | Y |
|  | Vilà, Carles, et al. "Rescue of a severely bottlenecked wolf (Canis lupus) population by a single immigrant." *Proceedings of the Royal Society of London B: Biological Sciences* 270.1510 (2003): 91-97. | Y | N |
|  | Vilà, Carles, et al. "Mitochondrial DNA phylogeography and population history of the grey wolf Canis lupus." *Molecular Ecology* 8.12 (1999): 2089-2103. | N | N |
|  | Mladenoff, David J., Theodore A. Sickley, and Adrian P. Wydeven. "Predicting gray wolf landscape recolonization: logistic regression models vs. new field data." *Ecological Applications* 9.1 (1999): 37-44. | Y | Y |
|  | Wayne, R. K., et al. "Conservation genetics of the endangered Isle Royale gray wolf." *Conservation Biology* 5.1 (1991): 41-51. | N | N |
|  | Liberg, Olof, et al. "Severe inbreeding depression in a wild wolf Canis lupus population." *Biology letters* 1.1 (2005): 17-20. | Y | N |
| Dingo | Glen, Al S., et al. "Evaluating the role of the dingo as a trophic regulator in Australian ecosystems." *Austral Ecology* 32.5 (2007): 492-501. | Y | N |
|  | Letnic, Mike, Euan G. Ritchie, and Christopher R. Dickman. "Top predators as biodiversity regulators: the dingo Canis lupus dingo as a case study." *Biological Reviews* 87.2 (2012): 390-413. | Y | N |
|  | Daniels, Mike J., and Laurie Corbett. "Redefining introgressed protected mammals: when is a wildcat a wild cat and a dingo a wild dog?." *Wildlife Research* 30.3 (2003): 213-218. | Y | N |
|  | Dickman, Chris R., Alistair S. Glen, and Mike Letnic. "Reintroducing the dingo: can Australia’s conservation wastelands be restored." *Reintroduction of top-order predators* 7 (2009): 238. | Y | N |
|  | Allen, Benjamin L., Richard M. Engeman, and Lee R. Allen. "Wild Dogma: An Examination Of Recent" Evidence" For Dingo Regulation Of Invasive Mesopredator Release In Australia." (2011). | Y | N |
|  | Elledge, Amanda E., et al. "An evaluation of genetic analyses, skull morphology and visual appearance for assessing dingo purity: implications for dingo conservation." *Wildlife Research* 35.8 (2009): 812-820. | N | N |
| Red wolf | Kalinowski, Steven T., Philip W. Hedrick, and Philip S. Miller. "No inbreeding depression observed in Mexican and red wolf captive breeding programs." *Conservation biology* 13.6 (1999): 1371-1377. | N | N |
|  | Miller, Craig R., Jennifer R. Adams, and Lisette P. Waits. "Pedigree‐based assignment tests for reversing coyote (Canis latrans) introgression into the wild red wolf (Canis rufus) population." *Molecular Ecology* 12.12 (2003): 3287-3301. | N | N |
|  | Phillips, Michael K., V. Gary Henry, and Brian T. Kelly. "Restoration of the red wolf." (2003). | Y | Y |
|  | Bohling, Justin H., and Lisette P. Waits. "Assessing the prevalence of hybridization between sympatric Canis species surrounding the red wolf (Canis rufus) recovery area in North Carolina." *Molecular Ecology* 20.10 (2011): 2142-2156. | Y | N |
|  | Brownlow, C. Alexander. "Molecular taxonomy and the conservation of the red wolf and other endangered carnivores." *Conservation Biology* 10.2 (1996): 390-396. | N | N |
|  | Kennerly, Erin, et al. "A gene expression signature of confinement in peripheral blood of red wolves (Canis rufus)." *Molecular Ecology* 17.11 (2008): 2782-2791. | N | N |
| Ethiopian wolf | Gottelli, Dada, et al. "Molecular genetics of the most endangered canid: the Ethiopian wolf Canis simensis." *Molecular Ecology* 3.4 (1994): 301-312. | Y | N |
|  | Sillero-Zubiri, C., A. A. King, and D. W. Macdonald. "Rabies and mortality in Ethiopian wolves (Canis simensis)." *Journal of Wildlife Diseases* 32.1 (1996): 80-86. | N | N |
|  | Haydon, D. T., M. K. Laurenson, and C. Sillero‐Zubiri. "Integrating epidemiology into population viability analysis: managing the risk posed by rabies and canine distemper to the Ethiopian wolf." *Conservation Biology* 16.5 (2002): 1372-1385. | N | N |
|  | Gottelli, Dada, et al. "The effect of the last glacial age on speciation and population genetic structure of the endangered Ethiopian wolf (Canis simensis)." *Molecular Ecology* 13.8 (2004): 2275-2286. | Y | Y |
|  | Marino, J., C. Sillero‐Zubiri, and D. W. Macdonald. "Trends, dynamics and resilience of an Ethiopian wolf population." *Animal Conservation* 9.1 (2006): 49-58. | Y | Y |
|  | Sillero-Zubiri, C., F. H. Tattersall, and D. W. Macdonald. "Habitat selection and daily activity of giant molerats Tachyoryctes macrocephalus: significance to the Ethiopian wolf Canis simensis in the Afroalpine ecosystem." *Biological Conservation* 72.1 (1995): 77-84. | Y | Y |
| Spotted hyena | Hayward, Matt W., and Gina J. Hayward. "Activity patterns of reintroduced lion Panthera leo and spotted hyaena Crocuta crocuta in the Addo Elephant National Park, South Africa." *African journal of ecology* 45.2 (2007): 135-141. | Y | N |
|  | Yirga, Gidey, et al. "Adaptability of large carnivores to changing anthropogenic food sources: diet change of spotted hyena (Crocuta crocuta) during Christian fasting period in northern Ethiopia." *Journal of Animal Ecology* 81.5 (2012): 1052-1055. | Y | Y |
|  | Holekamp, Kay E., and Stephanie M. Dloniak. "Intraspecific variation in the behavioral ecology of a tropical carnivore, the spotted hyena." *Advances in the Study of Behavior* 42 (2010): 189-229. | Y | Y |
|  | Abay, Gidey Yirga, et al. "Peri-urban spotted hyena (Crocuta crocuta) in northern Ethiopia: diet, economic impact, and abundance." *European Journal of Wildlife Research* 57.4 (2011): 759-765. | Y | Y |
|  | Yirga, Gidey, et al. "Spotted hyena (Crocuta crocuta) coexisting at high density with people in Wukro district, northern Ethiopia." *Mammalian Biology-Zeitschrift für Säugetierkunde* 78.3 (2013): 193-197. | Y | Y |
|  | Bout, Nicolas, Céline Born, and Colin Spohr. "Evidence that the spotted hyena is present in the rainforest-savannah mosaic of south-east Gabon." *Mammalian Biology-Zeitschrift für Säugetierkunde* 75.2 (2010): 175-179. | Y | Y |
| Dhole | Iyengar, Ararti, et al. "Phylogeography, genetic structure, and diversity in the dhole (Cuon alpinus)." *Molecular Ecology* 14.8 (2005): 2281-2297. | Y | Y |
|  | Volodina, Elena V., et al. "Biphonation may function to enhance individual recognition in the dhole, Cuon alpinus." *Ethology* 112.8 (2006): 815-825. | Y | N |
|  | Borah, Jimmy, et al. "Food habits of dholes (Cuon alpinus) in Satpura Tiger Reserve, Madhya Pradesh, India." *Mammalia* 73.2 (2009): 85-88. | Y | Y |
|  | Kamler, Jan F., et al. "The diet, prey selection, and activity of dholes (Cuon alpinus) in northern Laos." *Journal of Mammalogy* 93.3 (2012): 627-633. | Y | Y |
|  | Jenks, Kate E., et al. "Mapping the distribution of dholes, Cuon alpinus (Canidae, Carnivora), in Thailand." *mammalia* 76.2 (2012): 175-184. | Y | Y |
|  | Srivathsa, Arjun, et al. "On a dhole trail: examining ecological and anthropogenic correlates of dhole habitat occupancy in the Western Ghats of India." *PloS one* 9.6 (2014): e98803. | Y | Y |
| African wild dog | Girman, Derek J., et al. "A molecular genetic analysis of social structure, dispersal, and interpack relationships of the African wild dog (Lycaon pictus)." *Behavioral Ecology and Sociobiology* 40.3 (1997): 187-198. | N | N |
|  | Courchamp, Franck, and David W. Macdonald. "Crucial importance of pack size in the African wild dog Lycaon pictus." *Animal Conservation* 4.02 (2001): 169-174. | Y | N |
|  | Monfort, S. L., et al. "Evaluating adrenal activity in African wild dogs (Lycaon pictus) by fecal corticosteroid analysis." *Journal of Zoo and Wildlife Medicine* (1998): 129-133. | N | N |
|  | Lindsey, Peter A., Johan T. Du Toit, and M. G. L. Mills. "Attitudes of ranchers towards African wild dogs Lycaon pictus: conservation implications on private land." *Biological Conservation* 125.1 (2005): 113-121. | Y | Y |
|  | Gusset, M., et al. "Human–wildlife conflict in northern Botswana: livestock predation by Endangered African wild dog Lycaon pictus and other carnivores." *Oryx* 43.01 (2009): 67-72. | Y | N |
|  | Creel, Scott, and Nancy Marusha Creel. "Six ecological factors that may limit African wild dogs, Lycaon pictus." *Animal Conservation* 1.01 (1998): 1-9. | Y | N |
| Eurasian lynx | KRAMER‐SCHADT, S. T. E. P. H. A. N. I. E., et al. "Fragmented landscapes, road mortality and patch connectivity: modelling influences on the dispersal of Eurasian lynx." *Journal of Applied Ecology* 41.4 (2004): 711-723. | Y | N |
|  | Schadt, Stephanie, et al. "Assessing the suitability of central European landscapes for the reintroduction of Eurasian lynx." *Journal of Applied Ecology* 39.2 (2002): 189-203. | Y | N |
|  | Herfindal, Ivar, et al. "Prey density, environmental productivity and home-range size in the Eurasian lynx (Lynx lynx)." *Journal of Zoology* 265.01 (2005): 63-71. | Y | N |
|  | Schadt, Stephanie, et al. "Rule-based assessment of suitable habitat and patch connectivity for the Eurasian lynx." *Ecological Applications* 12.5 (2002): 1469-1483. | Y | N |
|  | Gaona, Pilar, Pablo Ferreras, and Miguel Delibes. "Dynamics and viability of a metapopulation of the endangered Iberian lynx (Lynx pardinus)." *Ecological monographs* 68.3 (1998): 349-370. | Y | N |
|  | Breitenmoser, Urs, et al. "Spatial organization and recruitment of lynx (Lynx lynx) in a re‐introduced population in the Swiss Jura Mountains." *Journal of Zoology* 231.3 (1993): 449-464. | Y | N |
| Sunda clouded leopard | Wilting, Andreas, et al. "Density of the Vulnerable Sunda clouded leopard Neofelis diardi in two commercial forest reserves in Sabah, Malaysian Borneo." *Oryx* 46.03 (2012): 423-426. | N | N |
|  | Brodie, Jedediah, and Anthony J. Giordano. "Density of the Vulnerable Sunda clouded leopard Neofelis diardi in a protected area in Sabah, Malaysian Borneo." *Oryx* 46.03 (2012): 427-430. | N | N |
|  | Christiansen, Per. "Species distinction and evolutionary differences in the clouded leopard (Neofelis nebulosa) and Diard's clouded leopard (Neofelis diardi)." *Journal of Mammalogy* 89.6 (2008): 1435-1446. | Y | N |
|  | Sollmann, Rahel, et al. "Bringing clarity to the clouded leopard Neofelis diardi: first density estimates from Sumatra." *Oryx* 48.04 (2014): 536-539. | Y | N |
| Clouded leopard | Kitchener, Andrew C., Mark A. Beaumont, and Douglas Richardson. "Geographical variation in the clouded leopard, Neofelis nebulosa, reveals two species." *Current Biology* 16.23 (2006): 2377-2383. | N | N |
|  | Howard, JoGayle, et al. "Successful ovulation induction and laparoscopic intrauterine artificial insemination in the clouded leopard (Neofelis nebulosa)." *Zoo Biology* 15.1 (1996): 55-69. | N | N |
|  | Austin, Sean C., et al. "Ecology and conservation of the leopard cat Prionailurus bengalensis and clouded leopard Neofelis nebulosa in Khao Yai National Park, Thailand." (2007): 1-14. | N | N |
|  | Christiansen, Per. "Species distinction and evolutionary differences in the clouded leopard (Neofelis nebulosa) and Diard's clouded leopard (Neofelis diardi)." *Journal of Mammalogy* 89.6 (2008): 1435-1446. | Y | N |
|  | Borah, Jimmy, et al. "Abundance and density estimates for common leopard Panthera pardus and clouded leopard Neofelis nebulosa in Manas National Park, Assam, India." *Oryx* 48.01 (2014): 149-155. | Y | N |
|  | Mohamad, Shariff Wan, et al. "The first description of population density and habitat use of the mainland clouded leopard Neofelis nebulosa within a logged-primary forest in South East Asia." *Population Ecology* 57.3 (2015): 495-503. | Y | N |
| Lion | Loveridge, A. J., et al. "The impact of sport-hunting on the population dynamics of an African lion population in a protected area." *Biological Conservation* 134.4 (2007): 548-558. | Y | Y |
|  | Saberwal, Vasant K., et al. "Lion‐human conflict in the Gir Forest, India." *Conservation Biology* 8.2 (1994): 501-507. | Y | Y |
|  | Bauer, H., and S. Van Der Merwe. "Inventory of free-ranging lions Panthera leo in Africa." *Oryx* 38.01 (2004): 26-31. | Y | N |
|  | Packer, Craig, et al. "Conservation biology: lion attacks on humans in Tanzania." *Nature* 436.7053 (2005): 927-928. | Y | Y |
|  | Packer, C., et al. "Effects of trophy hunting on lion and leopard populations in Tanzania." *Conservation Biology* 25.1 (2011): 142-153. | Y | Y |
|  | CREEL, SCOTT, and NANCY CREEL. "Lion density and population structure in the Selous Game Reserve: evaluation of hunting quotas and offtake." *African Journal of Ecology* 35.2 (1997): 83-93. | Y | N |
| Jaguar | Silver, Scott C., et al. "The use of camera traps for estimating jaguar Panthera onca abundance and density using capture/recapture analysis." *Oryx* 38.02 (2004): 148-154. | Y | Y |
|  | Sanderson, Eric W., et al. "Planning to save a species: the jaguar as a model." *Conservation Biology* 16.1 (2002): 58-72. | Y | Y |
|  | Eizirik, Eduardo, et al. "Phylogeography, population history and conservation genetics of jaguars (Panthera onca, Mammalia, Felidae)." *Molecular Ecology* 10.1 (2001): 65-79. | Y | N |
|  | Maffei, Leonardo, Erika Cuéllar, and Andrew Noss. "One thousand jaguars (Panthera onca) in Bolivia's Chaco? Camera trapping in the Kaa‐Iya National Park." *Journal of Zoology* 262.3 (2004): 295-304. | Y | N |
|  | Rabinowitz, Alan, and Kathy A. Zeller. "A range-wide model of landscape connectivity and conservation for the jaguar, Panthera onca." *Biological conservation* 143.4 (2010): 939-945. | Y | N |
|  | Conforti, Valeria Amorim, and Fernando Cesar Cascelli de Azevedo. "Local perceptions of jaguars (Panthera onca) and pumas (Puma concolor) in the Iguacu National Park area, south Brazil." *Biological Conservation* 111.2 (2003): 215-221. | Y | Y |
| Leopard | Hayward, M. W., et al. "Prey preferences of the leopard (Panthera pardus)." *Journal of Zoology* 270.2 (2006): 298-313. | Y | Y |
|  | Balme, Guy, Luke Hunter, and Rob Slotow. "Feeding habitat selection by hunting leopards Panthera pardus in a woodland savanna: prey catchability versus abundance." *Animal Behaviour* 74.3 (2007): 589-598. | Y | Y |
|  | Ramakrishnan, Uma, Richard G. Coss, and Neil W. Pelkey. "Tiger decline caused by the reduction of large ungulate prey: evidence from a study of leopard diets in southern India." *Biological Conservation* 89.2 (1999): 113-120. | Y | Y |
|  | Packer, C., et al. "Effects of trophy hunting on lion and leopard populations in Tanzania." *Conservation Biology* 25.1 (2011): 142-153. | Y | Y |
|  | Miththapala, Sriyanie, John Seidensticker, and Stephen J. O'Brien. "Phylogeographic subspecies recognition in leopards (Panthera pardus): molecular genetic variation." *Conservation Biology* 10.4 (1996): 1115-1132. | N | N |
|  | Marker, L. L., and A. J. Dickman. "Factors affecting leopard () spatial ecology, with particular reference to Namibian farmlands." *South African Journal of Wildlife Research* 35.2 (2005): 105-115. | Y | Y |
| Tiger | Karanth, K. Ullas, and James D. Nichols. "Estimation of tiger densities in India using photographic captures and recaptures." *Ecology* 79.8 (1998): 2852-2862. | Y | Y |
|  | O'Brien, Timothy G., Margaret F. Kinnaird, and Hariyo T. Wibisono. "Crouching tigers, hidden prey: Sumatran tiger and prey populations in a tropical forest landscape." *Animal Conservation* 6.02 (2003): 131-139. | Y | Y |
|  | Karanth, K. Ullas, et al. "Assessing tiger population dynamics using photographic capture-recapture sampling." *Ecology* 87.11 (2006): 2925-2937. | Y | Y |
|  | Luo, Shu-Jin, et al. "Phylogeography and genetic ancestry of tigers (Panthera tigris)." *PLoS Biol* 2.12 (2004): e442. | N | N |
|  | Linkie, Matthew, et al. "Assessing the viability of tiger subpopulations in a fragmented landscape." *Journal of Applied Ecology* 43.3 (2006): 576-586. | Y | Y |
|  | Wikramanayake, Eric D., et al. "An ecology‐based method for defining priorities for large mammal conservation: the tiger as case study." *Conservation Biology* 12.4 (1998): 865-878. | Y | Y |
| Snow leopard | Mishra, Charudutt, et al. "The role of incentive programs in conserving the snow leopard." *Conservation Biology* 17.6 (2003): 1512-1520. | Y | Y |
|  | Oli, Madan K., Iain R. Taylor, and M. Elizabeth Rogers. "Snow leopard Panthera uncia predation of livestock: an assessment of local perceptions in the Annapurna Conservation Area, Nepal." *Biological Conservation* 68.1 (1994): 63-68. | Y | Y |
|  | Jackson, Rodney M., et al. "Estimating Snow Leopard Population Abundance Using Photography and Capture‐Recapture Techniques." *Wildlife Society Bulletin* 34.3 (2006): 772-781. | Y | N |
|  | Bagchi, S., and C. Mishra. "Living with large carnivores: predation on livestock by the snow leopard (Uncia uncia)." *Journal of Zoology* 268.3 (2006): 217-224. | Y | Y |
|  | Oli, M. K., I. R. Taylor, and D. ME Rogers. "Diet of the snow leopard (Panthera uncia) in the Annapurna Conservation Area, Nepal." *Journal of Zoology* 231.3 (1993): 365-370. | Y | N |
|  | Lovari, S., et al. "Restoring a keystone predator may endanger a prey species in a human‐altered ecosystem: the return of the snow leopard to Sagarmatha National Park." *Animal Conservation* 12.6 (2009): 559-570. | Y | Y |
| Puma | Kelly, Marcella J., et al. "Estimating puma densities from camera trapping across three study sites: Bolivia, Argentina, and Belize." *Journal of Mammalogy* 89.2 (2008): 408-418. | Y | Y |
|  | Franklin, William L., et al. "Ecology of the Patagonia puma Felis concolor patagonica in southern Chile." *Biological Conservation* 90.1 (1999): 33-40. | Y | N |
|  | Conforti, Valeria Amorim, and Fernando Cesar Cascelli de Azevedo. "Local perceptions of jaguars (Panthera onca) and pumas (Puma concolor) in the Iguacu National Park area, south Brazil." *Biological Conservation* 111.2 (2003): 215-221. | Y | Y |
|  | Ernest, Holly B., et al. "Genetic structure of mountain lion (Puma concolor) populations in California." *Conservation Genetics* 4.3 (2003): 353-366. | Y | Y |
|  | Novack, Anthony J., et al. "Foraging ecology of jaguar (Panthera onca) and puma (Puma concolor) in hunted and non-hunted sites within the Maya Biosphere Reserve, Guatemala." *Journal of Zoology* 267.02 (2005): 167-178. | Y | Y |
|  | Palmeira, Francesca BL, et al. "Cattle depredation by puma (Puma concolor) and jaguar (Panthera onca) in central-western Brazil." *Biological conservation* 141.1 (2008): 118-125. | Y | N |

**
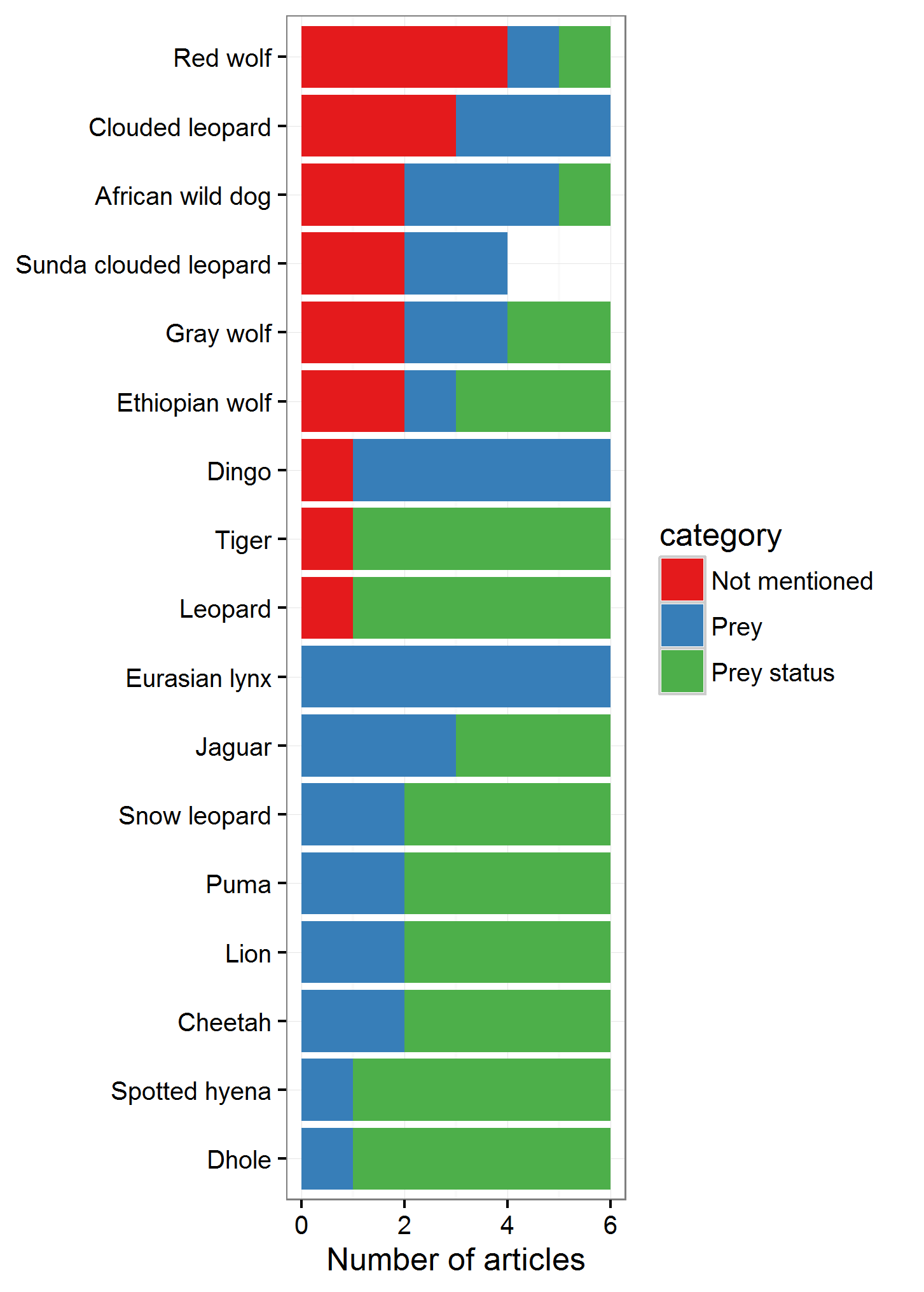
**

**Figure S12.** References to prey depletion in the literature. Results shown are for the top six most cited conservation articles for each large carnivore. Each article was categorized based on whether the term prey was not mentioned (“Not mentioned”), mentioned without reference to prey status or depletion (“Prey”), or mentioned in the context of prey status/depletion (“Prey status”).

**
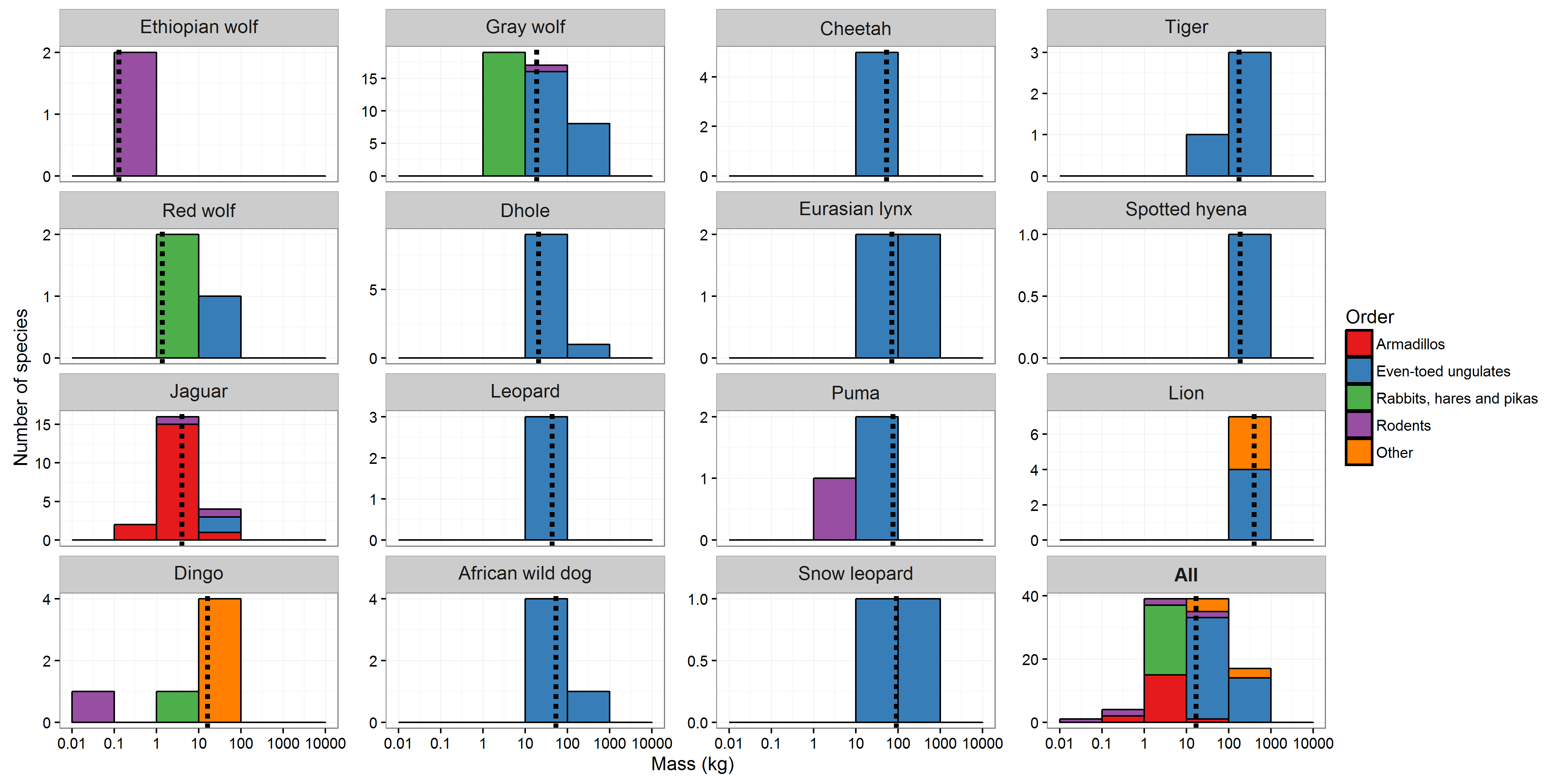
**

**Figure S13.** Mass and order information for each large carnivore’s preferred prey species. Results for all preferred prey species together are shown in the bottom right panel. Masses were obtained for 101 (89%) of the 114 total preferred prey species from Jones et al. (2009). Only orders with more than five preferred prey species appearing in large carnivore preferred prey lists are labeled. Carnivores are sorted top to bottom, left to right by median preferred prey mass (indicated by vertical lines).

Jones KE, Bielby J, Cardillo M, Fritz SA, O’Dell J, Orme CDL, et al. PanTHERIA: a species-level database of life history, ecology, and geography of extant and recently extinct mammals. Ecology. 2009 Aug 17;90(9):2648–2648.
